# Comparing Statistical Tests for Differential Network Analysis of Gene Modules

**DOI:** 10.3389/fgene.2021.630215

**Published:** 2021-05-19

**Authors:** Jaron Arbet, Yaxu Zhuang, Elizabeth Litkowski, Laura Saba, Katerina Kechris

**Affiliations:** ^1^Department of Biostatistics and Informatics, Colorado School of Public Health, University of Colorado Anschutz Medical Campus, Aurora, CO, United States; ^2^Department of Epidemiology, Colorado School of Public Health, University of Colorado Anschutz Medical Campus, Aurora, CO, United States; ^3^Department of Pharmaceutical Sciences, Skaggs School of Pharmacy and Pharmaceutical Sciences, University of Colorado Anschutz Medical Campus, Aurora CO, United States

**Keywords:** differential network analysis, differentially co-expressed modules, gene co-expression networks, statistical inference, networks

## Abstract

Genes often work together to perform complex biological processes, and “networks” provide a versatile framework for representing the interactions between multiple genes. Differential network analysis (DiNA) quantifies how this network structure differs between two or more groups/phenotypes (e.g., disease subjects and healthy controls), with the goal of determining whether differences in network structure can help explain differences between phenotypes. In this paper, we focus on gene co-expression networks, although in principle, the methods studied can be used for DiNA for other types of features (e.g., metabolome, epigenome, microbiome, proteome, etc.). Three common applications of DiNA involve (1) testing whether the connections to a single gene differ between groups, (2) testing whether the connection between a *pair* of genes differs between groups, or (3) testing whether the connections within a “module” (a subset of 3 or more genes) differs between groups. This article focuses on the latter, as there is a lack of studies comparing statistical methods for identifying differentially co-expressed modules (DCMs). Through extensive simulations, we compare several previously proposed test statistics and a new p-norm difference test (PND). We demonstrate that the true positive rate of the proposed PND test is competitive with and often higher than the other methods, while controlling the false positive rate. The R package discoMod (differentially co-expressed modules) implements the proposed method and provides a full pipeline for identifying DCMs: clustering tools to derive gene modules, tests to identify DCMs, and methods for visualizing the results.

## Introduction

Gene expression studies measure expression levels on thousands of genes, with a goal of identifying individual genes or groups of genes that explain differences between phenotypes of interest (e.g., disease subjects and healthy controls). An extensive literature exists regarding methods for identifying individual genes whose *mean* expression differs between groups (Soneson and Delorenzi, 2013; Huang et al., 2015), often referred to as differentially expressed genes. Pathway analysis (Huang et al., 2009; Emmert-Streib and Glazko, 2011; Ramanan et al., 2012; De Leeuw et al., 2016) aims to identify groups of genes (pathways or gene sets) that are enriched with differentially expressed genes (competitive tests) or whose overall mean structure differs between groups (self-contained tests). However, all of these methods ignore interactions between multiple genes.

In recent years, there is a growing interest in systems or network biology (Barabasi and Oltvai, 2004; Chuang et al., 2010; Barabási et al., 2011) in which one uses a statistical network to model the relationships between multiple genes (or other molecular features). For analyzing networks of gene expression (gene co-expression networks), genes are represented as nodes in the network, with the relationships between genes represented as lines/edges connecting the nodes. The strength of the connections is usually represented by a correlation matrix that measures the pairwise correlations between all genes. An adjacency matrix and the topological overlap measure (TOM) are other common forms of representing the connections between genes (Zhang and Horvath, 2005). See Singh et al. (2018) and van Dam et al. (2018) for review of important terminology and concepts used in gene co-expression network analysis.

In differential network analysis (DiNA), the goal is to determine whether the network structure differs between two or more phenotype groups (see de la Fuente, 2010; Kayano et al., 2014; Singh et al., 2018; Shojaie, 2020 for review). Many of the methods of DiNA of gene co-expression networks can be classified into three categories: (1) Identifying a single node (gene) in the network where the connections at that node differ between phenotype groups. For example (Lichtblau et al., 2017), compare 10 methods for quantifying node specific differences between groups. (2) Identifying *pairs* of genes whose correlation differs between two or more groups (Liu et al., 2010; Dawson et al., 2012; Fukushima, 2013; Ha et al., 2015; McKenzie et al., 2016; Siska et al., 2016), i.e., the focus is on the connection between only two genes at a time. (3) The last category, and the focus of this paper, attempts to identify subsets of co-expressed genes, called modules (also referred to as clusters or communities; Petereit et al., 2016) whose connections differ between phenotypes (Watson, 2006; Choi and Kendziorski, 2009; Gill et al., 2010; Tesson et al., 2010; Langfelder et al., 2011; Rahmatallah et al., 2014; Jardim et al., 2019). Modules are groups of multiple genes that interact in a coordinated manner, e.g., their expression levels are correlated. Two main approaches are used for defining modules: one may obtain *a priori* predefined modules from a database (e.g., KEGG, Kanehisa and Goto, 2000; GO, Ashburner et al., 2000), or one can use clustering methods (Langfelder and Horvath, 2008; Andreopoulos et al., 2009; Tesson et al., 2010; Xu and Wunsch, 2010) to derive data dependent modules. Comparing clustering methods for deriving data-dependent modules is beyond the scope of this paper (see Kakati et al., 2019 for one comparative study). After defining the modules, the final step is to test whether a module’s connections differ between phenotype groups, which is known as a “differentially co-expressed module” (DCM). The null hypothesis is that the network structure within the module is equal between the groups being compared. Although several methods have been proposed for testing whether the network structure within a module differs between two groups (Watson, 2006; Choi and Kendziorski, 2009; Gill et al., 2010; Tesson et al., 2010; Langfelder et al., 2011; Rahmatallah et al., 2014; Jardim et al., 2019), there is a lack of simulation studies comparing such methods. Therefore, we attempt to fill this gap by conducting extensive simulations of different network structures to compare existing test statistics for identifying DCMs, as well as a new framework the p-norm difference (PND) test that encompasses previous approaches but also provides more flexibility. Tests in the PND framework demonstrate a true positive rate that is competitive with and often higher than existing methods, while controlling the false positive rate. Lastly, the discoMod R package is made available, which implements a full pipeline for identifying DCMs: clustering tools to derive modules, tests to identify DCMs, and methods to visualize the results.

## Materials and Methods

Assume one has a list of *M* number of gene modules, which may have been predefined from a database (Ashburner et al., 2000; Kanehisa and Goto, 2000) or derived using clustering methods (Langfelder and Horvath, 2008; Andreopoulos et al., 2009; Tesson et al., 2010; Xu and Wunsch, 2010). Each module contains three or more genes, and the modules need not be disjoint (e.g., the same gene could appear in more than one module). Although we focus on genes, all the methods discussed can be used for other types of features besides gene expression (e.g., metabolome, epigenome, microbiome, proteome).

Let *X*^(*gm*)^ be the gene expression matrix for groups *g* = 1,2 and modules *m* = 1,…,*M*, where each gene expression variable may be measured as an integer count (i.e., number of mapped reads) from a sequencing platform or a continuous value from a microarray platform. Next, let *S*^(*gm*)^ be a similarity matrix used to represent the network structure of the *m*th module within the *g*th group. Note *S*^(*gm*)^ is a symmetric |*P*_*m*_|^*^|*P*_*m*_| matrix where |*P*_*m*_| represents the number of genes in the *m*th module, *i* = 1,2,…|*P*_*m*_| is the gene index, and $S_{i\,j}^{\left(g\,m\right)}$ is a measure of similarity between genes *i* and *j*. Several measures of similarity between two genes ($S_{i\,j}^{\left(g\,m\right)}$) have been used, including: correlation (Pearson, Spearman, or Kendall), partial correlation, or mutual information (Gill et al., 2010; Kumari et al., 2012; Kayano et al., 2014; van Dam et al., 2018). This similarity matrix may be further represented as an adjacency or TOM matrix (Ravasz et al., 2002; Zhang and Horvath, 2005; Langfelder and Horvath, 2008) which will be discussed later.

For the *m*th module with similarity matrices *S*^(*gm*)^ for both groups (*g* = 1,2), we are interested in testing the following null (*H*_0_) and alternative (*H_A_*) hypotheses:

(1) $$H_{0}:\;S^{\left(1m\right)}\;=S^{\left(2m\right)}\;vs.\;H_{A}:\;S^{\left(1m\right)}\neq S^{\left(2m\right)}$$

### Test Statistics for Identifying DCMs

We now define several test statistics that will be compared for testing (1). Given that *S*^(*gm*)^ is a symmetric |*P*_*m*_|^*^|*P*_*m*_| matrix, let *V*^(*gm*)^ be a vector of the lower triangle of *S*^(*gm*)^, thus *V*^(*gm*)^ is a vector of length $\lambda_{m}=\frac{\left|P_{m}\right|\,\left(\left|P_{m}\right|-1\right)}{2}.$ Let *k* = 1,…, λ_*m*_ be the indexing variable for iterating between the elements of *V*^(*gm*)^. Many test statistics can be formulated as functions of the difference (or product) in *V*^(*gm*)^ between the two groups. For example, the “Dispersion Index” (DI), used by GSCA (Choi and Kendziorski, 2009) and DiffCoEx (Tesson et al., 2010), for the *m*th module is defined as:

(2) $$D\,I\,\left(V^{\left(1\,m\right)},V^{\left(2\,m\right)}\right)=\sqrt{\frac{1}{\lambda_{m}}\,\sum_{k=1}^{\lambda_{m}}{\left(V_{k}^{\left(1\,m\right)}-V_{k}^{\left(2\,m\right)}\right)}^{2}}$$

The mean absolute difference (MAD) (Gill et al., 2010; Ruan et al., 2015), is defined as:

(3) $$M\,A\,D\,\left(V^{\left(1\,m\right)},V^{\left(2\,m\right)}\right)=\frac{1}{\lambda_{m}}\,\sum_{k=1}^{\lambda_{m}}\left|V_{k}^{\left(1\,m\right)}-V_{k}^{\left(2\,m\right)}\right|$$

The DGCA R package (McKenzie et al., 2016) simply uses the mean (or median) of the differences. A potential problem with this approach is that positive and negative differences can cancel out, thus losing power to detect DCMs where some correlations increase while other correlations decrease between conditions. Nevertheless, similar to their approach, we consider the paired t-test statistic (mean of the differences divided by the standard error of the mean difference):

(4) $$pairedT\left(V^{\left(1\,m\right)},V^{\left(2\,m\right)}\right)=\left[\frac{1}{\lambda_{m}}\sum_{k=1}^{\lambda_{m}}\left(V_{k}^{\left(1\,m\right)}-V_{k}^{\left(2\,m\right)}\right)\right]*\frac{\sqrt{\lambda_{m}}}{\sqrt{\frac{1}{\lambda_{m}}\,\sum_{k=1}^{\lambda_{m}}{\left(V_{k}^{\left(1\,m\right)}-V_{k}^{\left(2\,m\right)}\right)}^{2}}}$$

Similar to the paired t-test statistic, we also consider the Wilcoxon signed rank test statistic, as implemented in the *wilcox.test* base R function (R Core Team, 2018). The Wilcoxon signed rank test statistic ranks the differences of |*V*^(1*m*)^−*V*^(2*m*)^| and then sums the ranks where the sign of (*V*^(1*m*)^−*V*^(2*m*)^) is positive.

Three additional statistics are compared that were also considered in (Ruan et al., 2015): the Quadratic Assignment Procedure (QAP), GCOR, and Generalized Hamming Distance (GHD). These statistics are defined as:

(5) $$Q\,A\,P\,\left(V^{\left(1\,m\right)},V^{\left(2\,m\right)}\right)=\frac{1}{\lambda_{m}}\,\sum_{k=1}^{\lambda_{m}}V_{k}^{\left(1\,m\right)}*V_{k}^{\left(2\,m\right)}$$

(6) $$G\,C\,O\,R\,\left(V^{\left(1\,m\right)},V^{\left(2\,m\right)}\right)=\sum_{k=1}^{\lambda_{m}}\left(V_{k}^{\left(1\,m\right)}-{\bar{V}}^{\left(1\,m\right)}\right)*\left(V_{k}^{\left(2\,m\right)}-{\bar{V}}^{\left(2\,m\right)}\right)$$

(7) $$G\,H\,D\,\left(V^{\left(1\,m\right)},V^{\left(2\,m\right)}\right)=\frac{1}{\lambda_{m}}\,\sum_{k=1}^{\lambda_{m}}{\left[\left(V_{k}^{\left(1\,m\right)}-{\bar{V}}^{\left(1\,m\right)}\right)-\left(V_{k}^{\left(2\,m\right)}-{\bar{V}}^{\left(2\,m\right)}\right)\right]}^{2}$$

Where ${\bar{V}}^{\left(1\,m\right)}$ and ${\bar{V}}^{\left(2\,m\right)}$ are the means of $V_{k}^{\left(1\,m\right)}$ and $V_{k}^{\left(2\,m\right)}$, respectively.

The test statistic from GSNCA (Rahmatallah et al., 2014) is also considered in this manuscript. GSNCA does not fit within the previously described framework of comparing the difference (or product) of the vectors *V*^(1*m*)^ and *V*^(2*m*)^, thus we refer the reader to the original paper for the formal definition. Nevertheless, GSNCA can still be used to test whether the network structure of a module differs between the two groups. Briefly, GSNCA assigns a weight vector to each group of length |*P*_*m*_| (one weight per gene) and the test statistic is the sum of the absolute differences of the weight vector between the two groups. The *i*th gene is given a weight *w_i_* that is proportional to the sum of the correlations between the *i*th gene with all other genes. Thus, a gene that is highly correlated with many other genes will be given a larger weight, which indicates the gene may have regulatory importance.

We propose a new class of test statistics for identifying DCMs, the p-norm difference test (“PND”), which uses the p-norm (or *L^P^*norm) of the differences between *V*^(1*m*)^ and *V*^(2*m*)^.

(8) $$P\,N\,D\,\left(V^{\left(1\,m\right)},V^{\left(2\,m\right)},p\right)={\left(\frac{1}{\lambda_{m}}\,\sum_{k=1}^{\lambda_{m}}{\left|V_{k}^{\left(1\,m\right)}-V_{k}^{\left(2\,m\right)}\right|}^{p}\right)}^{\frac{1}{p}}$$

The motivation of the PND test is, given a “partially differentially co-expressed module” (a module where some of the correlations, but not all, change between groups), then the higher the exponent *p*, the less weight is given to the null correlations that do not change between groups. Therefore, we expect the PND test with a large value of *p* (e.g., *p* ≥ 4) to be more sensitive for detecting DCMs where only a small proportion of the module correlations change between conditions. In our simulations, we consider four different values for the exponent *p*: 4, 6, 8, and 20. Note the Dispersion Index is equivalent to the PND test with *p* = 2.

For all previously defined test statistics, the elements of *V*^(*gm*)^ are unlikely to be independent since they come from a structured similarity matrix (e.g., a correlation matrix), thus it is challenging to derive the sampling distribution under the null hypothesis without imposing additional assumptions. Therefore, we use a non-parametric permutation method to calculate p-values, which accounts for this complex dependency structure. Specifically, given a test statistic θ_*m*_ for the *m*th module, the permutation p-value is defined as follows:

1. Using the original gene expression matrices for each group, *X*^(1*m*)^ and *X*^(2*m*)^, and for a particular similarity measure of interest, calculate the similarity matrices *S*^(1*m*)^ and *S*^(2*m*)^. Then calculate the test statistic θ^*m*^ for testing the null hypothesis in (1)

2. For *b* = 1,2,…,*B* (*B* total number of permutations):

a. Combine the gene expression matrices of both groups, *X*^(1*m*)^ and *X*^(2*m*)^, and randomly shuffle (permute) the group labels, to create new “permuted” gene expression matrices *X*^(1*m*)^(*b*) and *X*^(2*m*)^(*b*).
b. Calculate the new similarity matrices *S*^(1*m*)^(*b*) and *S*^(2*m*)^(*b*) based on the permuted gene expression matrices *X*^(1*m*)^(*b*) and *X*^(2*m*)^(*b*)
c. Calculate the permuted test statistic θ^*m*^(*b*) based on *S*^(1*m*)^(*b*) and *S*^(2*m*)^(*b*).

3. Calculate the permutation *p*-value = $\frac{\left[\sum_{b=1}^{B}I\left(|\theta^{m}\left(b\right)|\geq |\theta^{m}|\right)\right]+1}{B+1}$

Lastly, we compare with three tests from the HDtest R package: HD (Chang et al., 2017), CLX (Cai et al., 2013), and Schott (Schott, 2007). These tests are designed to compare a high dimensional covariance matrix between two groups. CLX and Schott use asymptotic approximations to calculate *p*-values (and are thus much faster than all other methods considered) while HD uses a multiplier bootstrap method.

### Similarity Measures for Constructing Test Statistics

For the test statistics we are comparing, one needs to decide what type of similarity measure will be used for $S_{i\,j}^{\left(g\,m\right)}$ (similarity between any two genes *i* and *j* in the *m*th module of group *g*), when testing the null hypothesis of (1). As previously mentioned, several similarity measures have been used in practice: correlation (Pearson, Spearman, or Kendall), partial correlation, mutual information (Gill et al., 2010; Kumari et al., 2012; Kayano et al., 2014; van Dam et al., 2018), and adjacency or TOM matrices (Zhang and Horvath, 2005; Langfelder and Horvath, 2008). Gaussian and semi/nonparametric graphical models have also been used to measure the conditional dependence between each pair of genes (i.e., partial correlations) (Friedman et al., 2008; Wang et al., 2016; Zhang et al., 2018; Shojaie, 2020). It is beyond the scope of this paper to compare all of these similarity measures for constructing networks.

This paper will focus on comparing two particular types of unconditional similarity measures: correlation vs. TOM (Ravasz et al., 2002; Langfelder and Horvath, 2008). For correlation, we will use Spearman’s correlation (rather than Pearson’s correlation), based on recommendations from other studies (Kumari et al., 2012; Siska and Kechris, 2017). When calculating the similarity between two genes, $S_{i\,j}^{\left(g\,m\right)}$, unconditional correlation only considers the relationship between the two genes *i* and *j*, while ignoring any shared relationships these genes might have with other genes. This is true for all of the aforementioned measures of similarity, except for partial correlation and TOM. In contrast to unconditional correlation, TOM captures shared relationships or “neighbors” between the two genes, as defined in Equation (9) (note the signed version of the adjacency measure, *a*_*ij*_, is defined in Langfelder and Horvath, 2008).

(9) $$TOM_{ij}\;=\;\frac{a_{ij}+\sum_{u\neq i,j}^{}a_{iu}a_{uj}}{\min\left(k_{i},\;k_{j}\right)+1-a_{ij}}$$

$$k_{i}=\sum_{u}a_{i\,u},a_{i\,j}=\left\{ \begin{array}{c} {\left|c\,o\,r\,r_{i\,j}\right|}^{\beta }\,\mathrm{if}\,\mathrm{unsigned}\\ {\left|\frac{1+c\,o\,r\,r_{i\,j}}{2}\right|}^{\beta }\,\mathrm{if}\,\mathrm{signed} \end{array}\right.$$

The intuition behind TOM is that if the two genes *i* and *j* are connected to a common set of genes, then the similarity between the two genes, $S_{i\,j}^{\left(g\,m\right)},$ should increase (i.e., the greater the number and strength of the connections that are shared by genes *i* and *j*, the larger the TOM value will be for those two genes). To calculate TOM, one must first calculate the correlation matrix, then convert to an adjacency matrix (*a*_*ij*_), and then calculate the TOM matrix. We used the WGCNA R package *adjacency* function with type = “signed”, power = 1, and the *TOMsimilarity* function with TOMType = “signed.” We used “signed” versions since we want to be able to detect correlations that change from positive to negative between groups, when calculating $\left(V_{k}^{\left(1\,m\right)}-V_{k}^{\left(2\,m\right)}\right)$ in Equation (2) (e.g., for unsigned versions, a correlation that changes from 0.5 to −0.5 would result in a $V_{k}^{\left(1\,m\right)}-V_{k}^{\left(2\,m\right)}=0$ which is undesirable when trying to measure differential co-expression). When constructing the test statistics, our motivation for comparing correlation versus TOM, is to assess whether there is any benefit to averaging over the connections with other genes “*u*,” as TOM does through the numerator term ∑_*u≠i*,*j*_*a*_*iu*_*a*_*uj*_. Thus, we keep the exponent β = 1 for both the correlation and TOM approaches. If we were to set β≠1, than it would be unclear whether any differences in results for correlation versus TOM were due to the exponent, or the neighborhood averaging, and we are interested in the latter. In summary, the motivation for comparing correlation vs. TOM for constructing test statistics is to determine whether TOM is more sensitive to detecting DCMs when the number (and strength) of the connections that are *shared* between genes changes between conditions (e.g., cases vs. controls).

### Simulations

Simulations were used to compare the false positive rate (FPR) and true positive rate (TPR) between all of the test statistics under consideration, under several simplified correlation structures. If methods do not perform well under these simple scenarios, then they may not perform well under more complex network structures. The *rmvnorm* function within the mvtnorm R package (Genz et al., 2020) was used to simulate modules. Specifically, when simulating a given module *m*, an *N*^∗^|*P*_*m*_| gene expression matrix *X*^(*gm*)^ (*N* subjects,|*P*_*m*_| genes) is simulated for the *g*th group from a multivariate normal distribution with a zero mean vector, and a |*P*_*m*_|^*^|*P*_*m*_| correlation matrix ∑^(*gm*)^(the variance of each gene equals 1).

#### Null Simulations

To assess the FPR, a variety of “null” simulations were conducted where modules are simulated such that the correlation matrix is identical between the two groups (i.e., ∑^(1*m*)^ = ∑^(2*m*)^). In Equation (10), two different correlation structures are considered for the null simulations: compound symmetric (“CS,” i.e., constant pairwise correlation “ρ” between genes), and an “AR1” correlation structure where the correlation between genes “ρ” decays exponentially as genes get further apart.

(10) $$\text{CS correlation}:\;\left(\begin{array}{ccccc} 1 & \text{ρ} & \text{ρ} & \ldots & \text{ρ}\\ \text{ρ} & 1 & \text{ρ} & \ldots & \text{ρ}\\ \text{ρ} & \text{ρ} & 1 & \ldots & \text{ρ}\\ \vdots & \vdots & \vdots & \ddots & \vdots \\ \text{ρ} & \text{ρ} & \text{ρ} & \ldots & 1 \end{array}\right),\;\text{AR}1\text{ correlation}:\;\left(\begin{array}{ccccc} 1 & \text{ρ} & {\text{ρ}}^{2} & \ldots & {\text{ρ}}^{P-1}\\ \text{ρ} & 1 & \text{ρ} & \ldots & {\text{ρ}}^{P-2}\\ {\text{ρ}}^{2} & \text{ρ} & 1 & \ldots & {\text{ρ}}^{P-3}\\ \vdots & \vdots & \vdots & \ddots & \vdots \\ {\text{ρ}}^{P-1} & {\text{ρ}}^{P-2} & {\text{ρ}}^{P-3} & \ldots & 1 \end{array}\right)$$

For the CS and AR1 null scenarios, the following parameter values are considered: ρ= 0.3 or 0.7, *N* = 25 samples per group, and *P* = 10, 50, or 100 genes within each module. For each setting, 1,000 modules are simulated, and 3,000 permutations are used to calculate *p*-values.

A final scenario is considered with a “hub gene” network structure, since hub genes are common in many biological applications (Zhang and Horvath, 2005). Here there is a single hub gene, where all other genes have a correlation of ρ = 0.7 with the hub gene. To allow for smaller transitive correlation, the correlation between non hub genes is 0.4 in all simulations. A larger sample size (50 or 100 per group) was used for the hub gene simulations, since a larger sample size was needed in the DCM simulations of section “CS Where Correlations Change Direction,” in order to have higher TPR to compare between methods. The goal for all of these null simulations is to determine whether each method can control the FPR at level 0.05.

#### DCM Simulations

The DCM simulation framework is similar to section “Null Simulations,” except now the correlation within a module differs between the two groups (∑^(1*m*)^≠∑^(2*m*)^). Specifically, ∑^(1*m*)^ is fixed as one of the correlation structures from section “Null Simulations” (CS, AR1, or hub), while ∑^(2*m*)^ changes a randomly selected proportion, γ, of the lower triangle of ∑^(1*m*)^ (the same changes are then made in the upper triangle to ensure the correlation matrix remains symmetric). We consider γ = 0.1, 0.4, or 0.7 to represent small, medium and large effects. Five scenarios are considered 1) CS correlation (ρ = 0.3 or 0.7) with a proportion of correlations, γ, dropped to zero; 2) AR1 (ρ = 0.7) correlation with a proportion of correlations dropped to zero; 3) CS correlation with a proportion of the correlations changed, γ, such that half of the changed correlations increase 50% while the other half decrease by 50%; 4) CS correlation (ρ = 0.5) with a proportion of correlations, γ, changed to −0.5; and 5) hub gene structure with a proportion, γ, of the hub gene correlations dropped to zero. For scenario 3, we set ρ = 0.5, thus for the subset of changed correlations, half of the correlations increase to 0.75, while half decrease to 0.25. The motivation for scenarios 1–2 is to compare performance between methods given a module with homogeneous (CS) vs. heterogeneous (AR1) correlations that drop to zero. The motivation for scenario 3 and 4 is to compare performance when the changed correlations increase/decrease, or when the correlations change sign. The motivation for scenario 5 is to simulate a module with very sparse changes between groups: i.e., only one row in the correlation matrix changes between groups (the hub gene correlations). Lastly, when changing the population correlation matrices between the two groups, the *make.positive.definite* function from the lqmm R package (Geraci, 2014) is used to ensure that the changed correlation matrix is positive definite, which is necessary in order to simulate the modules from a multivariate normal distribution.

#### Case Study: Leukemia Microarray Data

All test statistics were compared using data from the leukemia microarray study of Golub et al. (1999). The dataset was downloaded from the multtest R package (Pollard et al., 2005), and contains tumor gene expression measured on 3051 genes from 27 subjects with acute lymphoblastic (ALL) and 11 subjects with acute myeloid leukemia (AML). The data was preprocessed according to Dudoit et al. (2002).

The mclust R package (Scrucca et al., 2016) was used to derive data driven modules within the ALL group, then the corresponding modules were obtained from the AML group and tested for differential co-expression. Then the process was repeated the other way: mclust was used to derive modules in the AML group, then the corresponding modules were obtained from the ALL group and tested for differential co-expression. This approach to module derivation is similar to that taken by CoXpress (Watson, 2006), however, CoXpress uses hierarchical clustering where the researcher must choose the height at which to cut the dendrogram, which determines the number of modules. In contrast, mclust uses the Bayesian Information Criterion (BIC) to determine the number of modules. Specifically, BIC was used to choose between two different diagonal cluster covariance structures (VII or EII), and to estimate the number of modules. The VII and EII covariance structures were chosen since they are the most parsimonious covariance structures included in mclust, which assume a diagonal covariance structure similar to k-means, but with the benefit of being able to use BIC to choose the number of modules. In addition, the assumption of a diagonal covariance structure has been shown to work well in other high dimensional supervised classification settings (Tibshirani et al., 2003; Bickel and Levina, 2004). After deriving the modules, 10,000 permutations were used to calculate *p*-values and false discovery rate (FDR) adjusted *p*-values were used to account for multiple testing (Benjamini and Hochberg, 1995). Example network graphics were generated using Cytoscape version 3.8.2 (Shannon et al., 2003).

## Results

A summary of all simulations settings is given in Supplementary Table 1.

### Null Simulations

Tables 1–3 present the false positive rate (FPR) of each method for the compound symmetric, AR1, and hub gene null simulations, respectively. For each simulation scenario, a one sample proportion test is used to assess whether the FPR of a given test statistic differs from the nominal rate of 0.05. For the one sample proportion tests, a *p*-value cutoff of 0.01 was used due to the large number of statistical tests. Overall, only the HD, CLX, and Schott methods were unable to control the FPR in some scenarios, thus these methods were removed from the DCM simulations of section “DCM Simulations,” in order to present a fair comparison of the true positive rates (TPR). For example, the maximum FPR observed was 16.9, 10.7, and 8.7% for the HD, CLX, and Schott tests, respectively. All other tests were able to control the FPR across all scenarios, and fluctuations from the nominal rate of 0.05 across *P* or ρ values are likely due to random variation.

**TABLE 1:** Compound symmetric null simulation false positive rates.

ρ	P	PND4	PND6	PND8	PND20	DI	MAD	pairedT	wilcoxSRT	GSNCA	GHD	QAP	GCOR	HD	CLX	Schott
0.3	10	0.045	0.051	0.047	0.048	0.051	0.049	0.058	0.053	0.049	0.050	0.055	0.059	0.077*	0.059	0.062
0.3	50	0.063	0.059	0.048	0.042	0.062	0.063	0.059	0.061	0.043	0.038	0.063	0.043	0.120*	0.073*	0.076*
0.3	100	0.060	0.063	0.062	0.047	0.060	0.058	0.057	0.055	0.049	0.054	0.052	0.050	0.169*	0.099*	0.087*
0.7	10	0.055	0.054	0.048	0.044	0.055	0.057	0.056	0.055	0.039	0.048	0.058	0.050	0.079*	0.026*	0.084*
0.7	50	0.038	0.037	0.040	0.046	0.037	0.039	0.040	0.043	0.052	0.045	0.051	0.054	0.077*	0.013*	0.070*
0.7	100	0.050	0.047	0.048	0.040	0.053	0.051	0.049	0.050	0.052	0.052	0.038	0.054	0.111*	0.019*	0.067

*All settings use N = 25 subjects per group.* ρ, compound symmetric correlation parameter; P, number of genes in each module. *False positive rate significantly differs from the nominal rate of 0.05 (one-sample proportion test p-value < 0.01).

**TABLE 2:** AR1 null simulation false positive rates.

ρ	P	PND4	PND6	PND8	PND20	DI	MAD	PairedT	wilcoxSRT	GSNCA	GHD	QAP	GCOR	HD	CLX	Schott
0.3	10	0.048	0.050	0.051	0.051	0.048	0.045	0.049	0.043	0.052	0.046	0.059	0.056	0.085*	0.069*	0.053
0.3	50	0.044	0.048	0.054	0.056	0.048	0.052	0.057	0.052	0.045	0.049	0.054	0.054	0.122*	0.094*	0.051
0.3	100	0.049	0.048	0.050	0.053	0.046	0.045	0.050	0.048	0.053	0.046	0.050	0.050	0.150*	0.107*	0.047
0.7	10	0.046	0.045	0.043	0.042	0.046	0.049	0.049	0.054	0.050	0.053	0.051	0.053	0.072*	0.045	0.063*
0.7	50	0.045	0.051	0.050	0.059	0.044	0.044	0.048	0.052	0.054	0.044	0.054	0.053	0.122*	0.079*	0.057
0.7	100	0.049	0.052	0.053	0.050	0.046	0.045	0.044	0.047	0.047	0.043	0.054	0.051	0.164*	0.105*	0.060

*All settings use N = 25 subjects per group.* ρ, AR1 correlation parameter; P, number of genes in each module. *False positive rate significantly differs from the nominal rate of 0.05 (one-sample proportion test p-value < 0.01).

**TABLE 3:** Hub gene null simulation false positive rates.

N	ρ	P	PND4	PND6	PND8	PND20	DI	MAD	pairedT	wilcoxSRT	GSNCA	GHD	QAP	GCOR	HD	CLX	Schott
50	0.7	10	0.038	0.040	0.044	0.043	0.040	0.040	0.049	0.055	0.049	0.051	0.053	0.048	0.054	0.035	0.082*
100	0.7	50	0.064	0.058	0.058	0.050	0.060	0.059	0.057	0.053	0.051	0.039	0.063	0.054	0.056	0.031*	0.084*

*N, number of subjects per group.* ρ, *correlation between the non-hub genes with the hub gene; P*, number of genes in the module. *False positive rate significantly differs from the nominal rate of 0.05 (one-sample proportion test p-value < 0.01).

### DCM Simulations

For each simulation setting, a line graph was used to compare the TPR between all methods for small, medium and large correlation effects (corresponding tables are found in Supplementary Material). The QAP and GCOR methods were removed from the line graphs to save space, since they consistently have the lowest TPR across all simulations.

#### CS With Correlations Dropped to Zero

**FIGURE 1:**
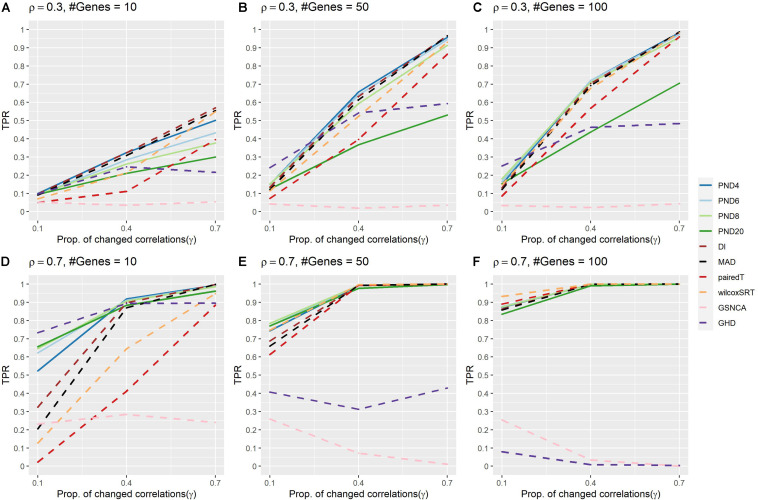
True positive rates for the compound symmetric DCM simulations with a proportion of correlations dropped to zero between the two groups. Solid lines refer to the proposed PND tests, while dashed lines refer to pre-existing methods. **(A–C)** Compound symmetric correlation parameter, ρ= 0.3, and the number of genes in a module (“#Genes”) is 10, 50, or 100. **(D–F)** Compound symmetric correlation parameter, ρ= 0.7, and the number of genes in a module (“#Genes”) is 10, 50, or 100. N=25 samples per group.

Figure 1 and Supplementary Table 2 present TPR for the compound symmetric DCM simulations where a proportion, γ, of the correlations are randomly changed to zero between the two groups. PND4 had a TPR within the top three highest TPR 13 times, followed by PND6 and DI (11), PND8 and MAD (9), with all other methods appearing in the top three at most 6 times. The QAP, GCOR and GSNCA methods consistently had the lowest TPRs, while PND20, MAD, pairedT, wilcoxSRT, and GHD methods were often more in the middle, with the GHD test having near zero TPR in Figure 1F. For most settings, there was little difference in TPR between PND4-8 and DI (note DI is equivalent to the PND with exponent 2, i.e., “PND2”). One exception was the tenth row of Supplementary Table 2 (ρ = 0.7, *P* = 10, γ = 0.1), where PND4 had 20% higher TPR than DI, while PND6-20 had ≥ 30% higher TPR than DI.

#### AR1 With Correlations Dropped to Zero

**FIGURE 2:**
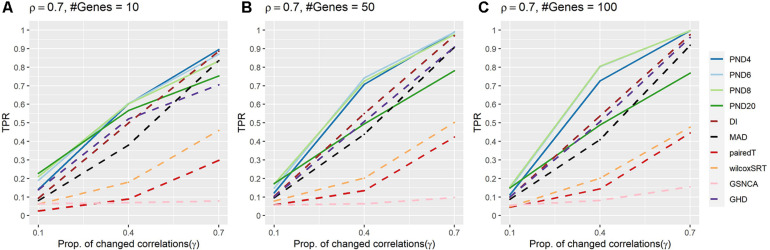
True positive rates for the AR1 DCM simulations with a proportion of correlations dropped to zero between the two groups. Solid lines refer to the proposed PND tests, while dashed lines refer to pre-existing methods. **(A–C)** AR1 correlation parameter, ρ = 0.7, and the number of genes in a module (“#Genes”) is 10, 50, or 100. N = 25 samples per group.

Figure 2 and Supplementary Table 3 present TPRs for the AR1 DCM simulations where a proportion, γ, of the correlations are randomly changed to zero between the two groups. In contrast to the previous CS simulations, the AR1 simulations consider a more heterogeneous set of population correlation values. In addition, the AR1 simulations have more separation in the TPR when comparing the PND tests with the DI and MAD tests. PND4-20 were in the top 3 highest TPRs 6, 9, 8, and 3 times, respectively, followed by DI which was in the top 3 one time. No other methods had TPR in the top three for any scenarios. PND4-8 were consistently near the top TPR, while PND20, DI, MAD, and GHD had TPR near the middle, with pairedT, wilcoxSRT, GSNCA, QAP, and GCOR consistently having the lowest TPR.

#### CS Where Half of the Changed Correlations Increase 50%, Half Decrease 50%

**FIGURE 3:**
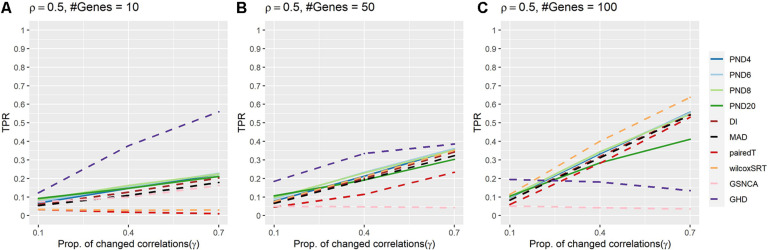
True positive rates for the compound symmetric DCM simulations with a proportion of correlations changed between groups such that half of the changed correlations increased by 50%, while the other half decreased by 50%. Solid lines refer to the proposed PND tests, while dashed lines refer to pre-existing methods. **(A–C)** Compound symmetric correlation parameter, ρ = 0.5, and the number of genes in a module (“#Genes”) is 10, 50, or 100. N = 25 samples per group.

Figure 3 and Supplementary Table 4 present TPRs for compound symmetric simulations where a proportion, γ, of the correlations are randomly changed such that half of the changed correlations increase by 50%, while the other half decrease by 50%. In contrast to previous sections, power was lower for most methods, with GHD as the most powerful test in Figures 3A,B and wilcoxSRT was the most powerful in Figure 3C. However, GHD became less powerful as the number of genes increased, with most other methods having TPR higher than GHD in Figure 3C. The GHD had TPR in the top 3 for 7 settings, followed by PND6 and PND8 (6), and three times for PND4, PND20, and wilcoxSRT.

#### CS Where Correlations Change Direction

Supplementary Figure 1 and Supplementary Table 5 present TPRs for compound symmetric (ρ = 0.5) simulations where a proportion of correlations, γ, of the correlations are randomly changed to –0.5. PND6 had TPR in the top 3 for all 9 settings, followed by PND8 (8), DI, MAD, wilcoxSRT (6). Similar to section “CS Where Half of the Changed Correlations Increase 50%, Half Decrease 50%,” the TPR for the GHD test substantially decreased as the number of genes increased. The PND4-8 tests, DI and MAD usually had the highest TPRs, with the PND tests having higher TPR when only 10% of the correlations were changed between groups.

#### Hub Gene Setting Where a Proportion of the Hub Gene Correlations Are Dropped to 0

**FIGURE 4:**
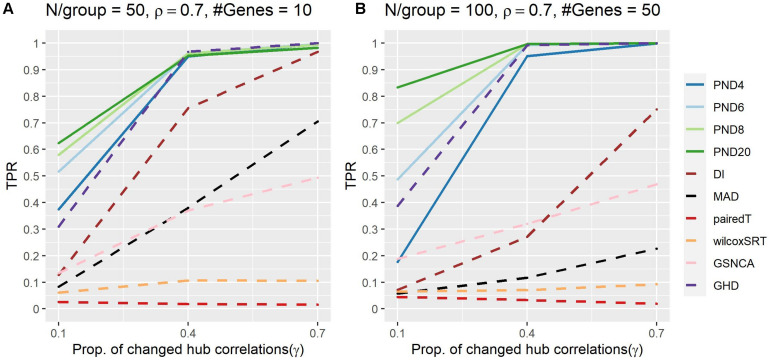
True positive rates shown for the hub gene DCM simulations with a proportion of hub-correlations dropped to zero; true positive rates shown for all methods. Solid lines refer to the proposed PND tests, while dashed lines refer to pre-existing methods. **(A–B)** N=50 or 100 samples per group, correlation between non hub-genes with the hub gene is ρ = 0.7, and the number of genes (“#Genes”) is 10 or 50.

Figure 4 and Supplementary Table 6 present TPRs for the hub gene correlation structure where a proportion, γ, of the hub gene correlations are dropped to zero. PND6 was in the top three highest TPRs 6 times, followed by PND8 (5), PND20 (4), GHD (3), and PND4 (1). No other methods had TPR in the top three for any scenarios. For most scenarios the PND and GHD tests have substantially higher TPR compared with DI, MAD, and the other remaining tests. Having a higher exponent in the PND tests (6 or higher) resulted in higher TPR compared to PMD4 when only 10% the hub gene correlations changed between the two groups.

#### Comparing Correlation Versus TOM Similarity Measures

As explained in section “Similarity Measures for Constructing Test Statistics,” we were interested in comparing two different similarity measures for constructing the test statistics: correlation versus the TOM. Given that TOM has a higher computational cost compared to correlation, results comparing with TOM are only shown for a subset of tests (PND6, DI, MAD, GHD) and simulation settings, using 100 simulation replicates and 2,000 permutations. Supplementary Figure 2 displays a line graph comparing the TPR in correlation-based methods (solid lines) to their TOM counterparts (dashed lines). In nearly all simulation settings, the TOM methods had lower TPR than their correlation counterparts. Two exceptions were: Supplementary Figure 2A when γ = 0.7, PND6 had slightly higher TPR when using TOM compared to correlation; and Supplementary Figure 2E where the TPR of GHD was higher when using TOM, particularly when γ = 0.1. Overall, given the increased computational cost of TOM, and the fact that TOM had lower TPR in nearly all simulation settings, the TOM based methods are omitted from the remainder of the paper.

#### Overall Comparison of Tests Across All Simulation Studies

In summary, we evaluated 51 different simulation scenarios and the median TPR across these scenarios was greater than 0.70 for all PND methods (Supplementary Table 7). The DI and MAD methods followed with median TPR of 0.63 and 0.54, respectively. As alternative summaries, we also examined which methods ranked in the top three of all methods based on highest TPR or whether their TPR was within 5% of the highest TPR value (Supplementary Table 7). Based on these metrics PND4, PND6 and PND8 were in these top lists 58–80% of the times, followed by PND20, DI, MAD, WilcoxSRT and GHD, which were in these top lists 25–51% of the time.

### Case Study: Leukemia Microarray Data

We used the Golub leukemia data set to illustrate the application of DiNA, in addition to the visualization of module results. Supplementary Table 8 reports the following information for each of 86 derived modules: number of genes, *p*-values and FDR adjusted *p*-values for a subset of the top performing tests from our simulations (PND6, DI, MAD, GHD). Note when deriving the modules in the ALL group, BIC selected 49 modules with the VII covariance structure. The median module size had 64 genes (25th and 75th quantiles: 35 and 79 genes). When deriving the modules in the AML group, BIC selected 37 modules with the EII covariance structure. The median module size had 34 genes (25th and 75th quantiles: 20 and 133 genes).

**FIGURE 5:**
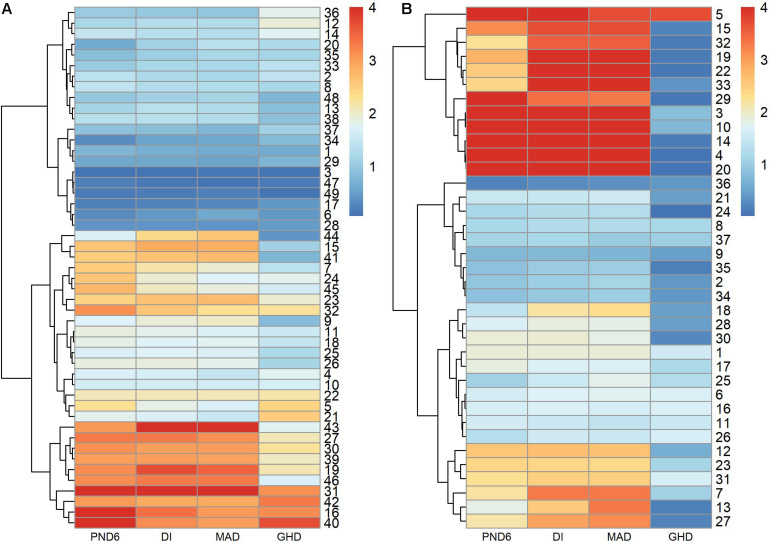
–log10 *p*-values of modules derived in leukemia microarray dataset. Hierarchical clustering was used to sort the modules and tests. **(A)** 49 modules derived in ALL group. **(B)** 37 modules derived in AML group.

Figures 5A,B compares the –log10 *p*-values among the PND6, DI, MAD, and GHD tests. The PND6, DI, and MAD tended to produce similar *p*-values, with GHD generally having larger *p*-values, especially for the AML derived modules. Supplementary Figure 3 presents a Venn diagram for the total number of modules with FDR adjusted *p*-values < 0.01 for each method. The DI and MAD methods had the most overlap with 9 modules that were only identified using these two methods. Of interest is that 2 modules were identified using the PND6 method only and 1 module that was only identified using the MAD method. For the nine modules only identified using the DI and MAD methods, the PND6 FDR ranged from 0.01 to 0.03 with unadjusted *p*-values well within the range of the unadjusted *p*-values for DI and MAD. Likewise, for the MAD only and the PND6 only modules, the FDR ranged from 0.01 to 0.04 for the other methods (not including GHD).

**FIGURE 6:**
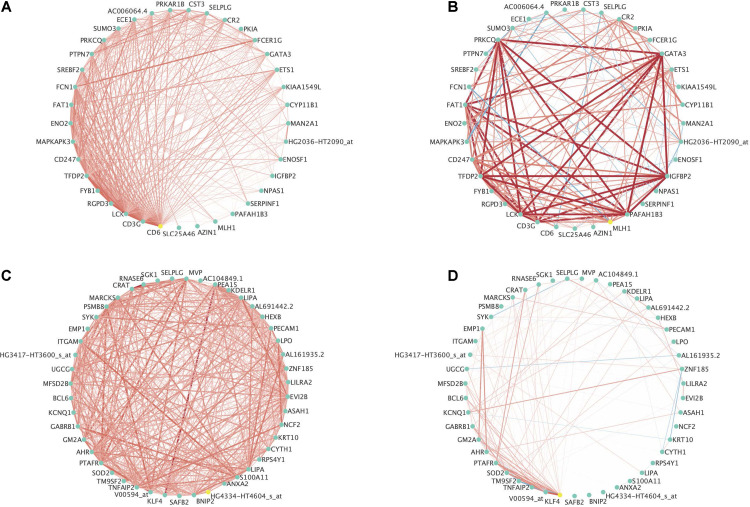
Example differentially expressed networks from the leukemia microarray data. For all networks, circles present individual probe sets. Labels are gene symbols for probe sets with annotation information in the Ensembl database. Otherwise, the original probe set identifier from Affymetrix was used. Red lines connecting circles indicate a positive correlation (correlation coefficient > 0.3) between the two probe sets. Blue lines connecting circles indicates a negative correlation between the two probe sets (correlation coefficient < –0.30). The intensity of the color and thickness of the lines are associated with the magnitude of the correlation between the two probe sets. **(A)** Associations between probe sets among the ALL subjects for Module 19 originally identified among the ALL subjects and **(B)** Associations between probe sets among the AML subjects for Module 19 originally identified among the ALL subjects. This module was significantly differentially co-expressed using the PND6 method (FDR < 0.01), the DI method (FDR < 0.01), and the MAD method (FDR < 0.01). It was borderline significant using the GHD method (FDR = 0.06). **(C)** Associations between probe sets within the AML subjects for Module 29 originally identified among the AML subjects and **(D)** Associations between probe sets within the ALL subjects for Module 29 originally identified among the AML subjects. This module was significantly differentially co-expressed using the PND6 method (FDR < 0.01), the DI method (FDR < 0.01), and the MAD method (FDR < 0.01). It was not significant with the GHD method (unadjusted *p*-value = 0.84; FDR = 0.90).

Figure 6 contains two examples of differential co-expression in this data. For ease of visualization, we focused on modules with less than 50 genes that were differentially expressed (FDR < 0.01) in at least three of the four methods explored. This resulted in 8 modules where all modules were differentially co-expressed using PND6, DI, and MAD and none were differentially co-expressed using the GHD method. The module among the 8 with the smallest *p*-value using the GHD method (ALL_19) and the module with the largest *p*-value using the GHD method (AML_29) were chosen for visualization. Figures 6A,B is a module that was identified in the ALL subjects that was differentially co-expressed in the AML subjects. In the original module (Figure 6A) all of the probe sets are positively correlated. Within the AML subjects, many of the correlations increased in intensity (light red to bright red), some correlations were dropped to approximately zero, and a few went from a positive association (red line) to a negative association (blue line). Figures 6C,D is a module that was originally identified in the AML subjects and was differentially co-expressed in the ALL subjects. For this module, most of the correlations among the probe sets dropped to values close to zero indicating a co-expression network that was only active in the AML group and not in the ALL group. See Supplementary Figures 4, 5 for correlation heatmaps as additional visualizations of differential co-expression in ALL_19, AML_29, and several other example modules with FDR adjusted *p*-values < 0.01.

Because PND6 did marginally better in the simulation studies, we further explored the module identified (FDR < 0.01) only when using the PND6 method whose unadjusted *p*-values in all other methods were greater than or equal to 0.01, ALL_24. Unlike in the modules depicted in Figure 6, the co-expression patterns of only a few genes in ALL_24 changed dramatically rather than all relationship, i.e., edges, changing in a coordinated way (Supplementary Figures 6A–C). To quantify this observation, we calculated the median difference in correlations for each gene. A large spread of the median difference between genes within a module would indicate connections for only few genes are changing dramatically, but most genes maintain their original connections (similar to simulation 5). When compared to a module that is not differentially co-expressed (ALL_3) and the two differentially co-expressed modules from Figure 6, the PND6 exclusive module, ALL_24, has a highly skewed distribution of median correlation differences, i.e., only associations with a few genes are dramatically altered (Supplementary Figure 6D). This trend held true among all modules as the ALL_24 had the largest estimated skewness among all modules (skewness = 2.87).

Within ALL_24, *cathepsin G* (*CTSG*) had the largest median difference (median difference = 0.90). Many of its edges changed from strong positive correlations to strong negative correlations among genes. CTSG is a well-established therapeutic target for both AML and ALL cancers (e.g., Jin et al., 2013; Khan et al., 2017). In a functional enrichment through EnrichR (Kuleshov et al., 2016), cellular response to cytokine stimulus (GO:0071345) was significantly enriched (adjusted *p*-value < 0.01) among the genes within ALL_24. Nine of the 59 genes within the module were associated with this GO term. Although CTSG was not associated directly with this GO term, its role in inflammation can easily be connected to the other genes (e.g., Gao et al., 2018). These results suggest that the role of CTSG in the inflammatory response to leukemia may differ between AML and ALL. Not only does this differentially co-expressed module indicate that this pattern of differential co-expression in present in “real” data, but it also indicates that this pattern can be biologically relevant.

## Discussion

Statistical networks provide a convenient framework for representing the interactions between multiple genes (or other molecular features). Differential network analysis (DiNA) quantifies how this network structure differs between two or more groups/phenotypes (e.g., disease subjects and healthy controls), and is a growing field of research (de la Fuente, 2010; Kayano et al., 2014; Singh et al., 2018; Shojaie, 2020). One major application of DiNA is to identify “modules” (subsets of 3 or more genes), where the network connections within a module differ between phenotype groups, known as differentially co-expressed modules (DCMs). Although several statistical tests have been proposed for identifying DCMs (Watson, 2006; Choi and Kendziorski, 2009; Gill et al., 2010; Tesson et al., 2010; Rahmatallah et al., 2014), there is a lack of simulation studies comparing such methods. Thus, the primary motivation of this study was to compare existing methods via simulations, as well as the proposed framework of the p-norm difference test (PND) which encompasses existing methods such as DI and MAD.

In the “Null Simulations” section (where the network structure within the module was identical between groups), all of the permutations based test statistics were able to control the FPR (PND4-20, DI, MAD, pairedT, wilcoxSRT, GSNCA, GHD, QAP, and GCOR). However, the three tests from the HDtest R package (CLX and Schott use asymptotic approximations to calculate *p*-values, while HD uses a “multiplier bootstrap” method) were often unable to control the FPR at level 0.05, and thus were omitted from the remainder of the manuscript. It is possible these methods may control the FPR given larger sample sizes, however, even with 50 or 100 samples per group (Table 3), the CLX and Schott tests did not control the FPR, although the HD test did control the FPR in these settings.

In the DCM simulations, it is worth noting that the TPRs of methods depend on the network structure. In the homogenous correlation structure of section “CS With Correlations Dropped to Zero”, the PND4-8, DI and MAD tests had the highest TPRs. In the more heterogeneous correlation structure of section “AR1 With Correlations Dropped to Zero”, there was greater separation in TPR when comparing PND4-20 with DI and MAD, with PND4-20 having the highest TPRs. In section “CS Where Half of the Changed Correlations Increase 50%, Half Decrease 50%”, the GHD test had the highest TPR in most settings, with the wilcoxSRT and PND tests surpassing the GHD as the number of genes increased. In the hub gene simulations of section “CS Where Correlations Change Direction,” the PND4-20 and GHD tests had the highest TPR.

Despite differences based on network structure, on average, test statistics in the PND framework were consistently the best performing (PND4-20, DI, MAD). In many of the scenarios, we found advantages of intermediate values for the power (e.g., PND 6 and 8) Thus for the question of what the exponent value should be for PND, we recommend PND6 as a default choice but recommend users to explore other power values based on their particular data sets.

One of the difficulties of evaluating differential co-expression techniques is to determine if the simulated scenarios are biologically relevant in any or all experimental designs. We have shown the existence of the hub gene framework in the AML/ALL case study. However, we did not observe patterns of differential co-expression in the AML/ALL dataset similar to all simulation scenarios. This observation does not indicate these simulation scenarios are not biologically relevant, they were just not observed under these experimental conditions.

This study is not without limitations, thus we identify five areas for future research. (1) Given that the TPRs of methods depends on the true network structure, it would be interesting to consider methods that combine multiple test statistics, in order to increase sensitivity across a greater variety of network structures. (2) Further research is needed for comparing other types of similarity measures for constructing the test statistics, such as various types of “conditional” partial correlation measures (Shojaie, 2020), or settings where using the TOM may improve power compared to correlation (3). Although one may use predefined modules from an existing database (e.g., KEGG, Kanehisa and Goto, 2000; GO; Ashburner et al., 2000), further research is needed to compare clustering methods for deriving data dependent modules, and determining the optimal number of modules. In section “Case Study: Leukemia Microarray Data,” we used a similar approach to CoXpress (Watson, 2006) but with model based clustering and BIC to select the number of modules. Instead of performing the clustering twice, as in CoXpress, DiffCoEx (Tesson et al., 2010) uses hierarchical clustering only once where the distance matrix is the TOM of the difference between two correlation matrices. WGCNA is another approach for deriving network modules using hierarchical clustering with a dynamic tree-cutting algorithm for choosing the number of modules (also used by DiffCoEx). However, the authors admit that it remains an open research question for how to optimize the tree-cutting parameters to determine the number of modules (Langfelder and Horvath, 2008; Langfelder et al., 2008). (4) This manuscript focused on comparing methods for identifying DCMs between two phenotype groups. Further research is needed for developing methods to identify DCMs for quantitative outcomes, or for categorical outcomes with more than two groups. (5) Lastly, more research is needed for differential network analysis when integrating multiple different types of molecular features (e.g., transcriptome, metabolome, microbiome, proteome). Some existing methods include: (Class et al., 2018; Erola et al., 2019; Shi et al., 2019).

In summary, several test statistics for identifying differentially co-expressed modules (DCMs) were compared via simulations and a leukemia microarray study (Golub et al., 1999). Through extensive simulations, tests in the PND framework had TPR that was competitive with and often higher than the other methods, while controlling the FPR. When comparing two different similarity measures for constructing the test statistics, correlation versus TOM, we found little benefit of using the more computationally expensive TOM. An approach to deriving data dependent modules was demonstrated using the dataset of (Golub et al., 1999), by using Gaussian mixture models with BIC to select the number of modules. However, further research is needed to compare clustering methods for deriving data dependent modules. Nevertheless, after obtaining a list of modules (predefined or data driven), we recommend the user take an intermediate power in the PND framework, such as PND6, for identifying DCMs. All methods considered are implemented in the discoMod R package, available at https://github.com/arbet003/discoMod.

## Data Availability Statement

Publicly available datasets were analyzed in this study. This data can be found here: https://www.bioconductor.org/packages/release/bioc/html/multtest.html.

## Author Contributions

JA and YZ further developed the project direction, wrote R code for simulations, analyzed the leukemia microarray dataset, and developed the discoMod R package. JA wrote the manuscript. KK and LS co-supervised this project. The thesis work of EL influenced the research questions and methods compared. All authors read and approved the final version of the manuscript, made substantial contributions to the conception, design, drafting, and revisions of this project.

## Conflict of Interest

The authors declare that the research was conducted in the absence of any commercial or financial relationships that could be construed as a potential conflict of interest.

## Abbreviations

DiNA: differential network analysis
DCM: differentially co-expressed module
TOM: topological overlap measure
PND: p-norm difference test
DI: dispersion index
MAD: mean absolute difference
paired: paired t-test statistic
wilcoxSRT: Wilcoxon signed rank test statistic
QAP: Quadratic assignment procedure test statistic
GHD: Generalized Hamming distance test statistic
TPR: true positive rate
FPR: false positive rate
CS: compound symmetric correlation structure
AR1: autoregressive order 1 correlation structure
BIC: Bayesian information criterion.

## References

[B1] Andreopoulos B. An A. Wang X. Schroeder M. (2009). A roadmap of clustering algorithms: finding a match for a biomedical application. *Brief. Bioinform.* 10 297–314. 10.1093/bib/bbn058 19240124

[B2] Ashburner M. Ball C. A. Blake J. A. Botstein D. Butler H. Cherry J. M. (2000). Gene ontology: tool for the unification of biology. *Nat. Genet.* 25 25–29. 10802651 10.1038/75556 PMC3037419

[B3] Barabási A.-L. Gulbahce N. Loscalzo J. (2011). Network medicine: a network-based approach to human disease. *Nat. Rev. Genet.* 12 56–68. 10.1038/nrg2918 21164525 PMC3140052

[B4] Barabasi A.-L. Oltvai Z. N. (2004). Network biology: understanding the cell’s functional organization. *Nat. Rev. Genet.* 5 101–113. 10.1038/nrg1272 14735121

[B5] Benjamini Y. Hochberg Y. (1995). Controlling the false discovery rate: a practical and powerful approach to multiple testing. *J. R. Stat. Soc. Series B Stat. Methodol.* 57 289–300. 10.1111/j.2517-6161.1995.tb02031.x

[B6] Bickel P. J. Levina E. (2004). Some theory for Fisher’s linear discriminant function, naive Bayes’, and some alternatives when there are many more variables than observations. *Bernoulli* 10 989–1010.

[B7] Cai T. Liu W. Xia Y. (2013). Two-sample covariance matrix testing and support recovery in high-dimensional and sparse settings. *J. Am. Stat. Assoc.* 108 265–277. 10.1080/01621459.2012.758041

[B8] Chang J. Zhou W. Zhou W. X. Wang L. (2017). Comparing large covariance matrices under weak conditions on the dependence structure and its application to gene clustering. *Biometrics* 73 31–41. 10.1111/biom.12552 27377648

[B9] Choi Y. Kendziorski C. (2009). Statistical methods for gene set co-expression analysis. *Bioinformatics* 25 2780–2786. 10.1093/bioinformatics/btp502 19689953 PMC2781749

[B10] Chuang H.-Y. Hofree M. Ideker T. (2010). A decade of systems biology. *Annu. Rev. Cell Dev. Biol.* 26 721–744. 10.1146/annurev-cellbio-100109-104122 20604711 PMC3371392

[B11] Class C. A. Ha M. J. Baladandayuthapani V. Do K.-A. (2018). iDINGO—integrative differential network analysis in genomics with Shiny application. *Bioinformatics* 34 1243–1245. 10.1093/bioinformatics/btx750 29194470 PMC6030922

[B12] Dawson J. A. Ye S. Kendziorski C. (2012). R/EBcoexpress: an empirical Bayesian framework for discovering differential co-expression. *Bioinformatics* 28 1939–1940. 10.1093/bioinformatics/bts268 22595207 PMC3492001

[B13] de la Fuente A. (2010). From ‘differential expression’to ‘differential networking’–identification of dysfunctional regulatory networks in diseases. *Trends Genet.* 26 326–333. 10.1016/j.tig.2010.05.001 20570387

[B14] De Leeuw C. A. Neale B. M. Heskes T. Posthuma D. (2016). The statistical properties of gene-set analysis. *Nat. Rev. Genet.* 17:353. 10.1038/nrg.2016.29 27070863

[B15] Dudoit S. Fridlyand J. Speed T. P. (2002). Comparison of discrimination methods for the classification of tumors using gene expression data. *J. Am. Stat. Assoc.* 97 77–87. 10.1198/016214502753479248 12611515

[B16] Emmert-Streib F. Glazko G. V. (2011). Pathway analysis of expression data: deciphering functional building blocks of complex diseases. *PLoS Comput. Biol.* 7:e1002053. 10.1371/journal.pcbi.1002053 21637797 PMC3102754

[B17] Erola P. Bonnet E. Michoel T. (2019). Learning differential module networks across multiple experimental conditions. *Methods Mol. Biol.* 1883 303–321. 10.1007/978-1-4939-8882-2_13 30547406

[B18] Friedman J. Hastie T. Tibshirani R. (2008). Sparse inverse covariance estimation with the graphical lasso. *Biostatistics* 9 432–441. 10.1093/biostatistics/kxm045 18079126 PMC3019769

[B19] Fukushima A. (2013). DiffCorr: an R package to analyze and visualize differential correlations in biological networks. *Gene* 518 209–214. 10.1016/j.gene.2012.11.028 23246976

[B20] Gao S. Zhu H. Zuo X. Luo H. (2018). Cathepsin G and its role in inflammation and autoimmune diseases. *Arch. Rheumatol.* 33 498–504. 10.5606/archrheumatol.2018.6595 30874236 PMC6409175

[B21] Genz A. Bretz F. Miwa T. Mi X. Leisch F. Scheipl F. (2020). Package ‘mvtnorm’. *J. Comput. Graphic. Stat.* 11 950–971.

[B22] Geraci M. (2014). Linear quantile mixed models: the lqmm package for Laplace quantile regression. *J. Stat. Softw.* 57 1–29.25400517

[B23] Gill R. Datta S. Datta S. (2010). A statistical framework for differential network analysis from microarray data. *BMC Bioinform.* 11:95. 10.1186/1471-2105-11-95 20170493 PMC2838870

[B24] Golub T. R. Slonim D. K. Tamayo P. Huard C. Gaasenbeek M. Mesirov J. P. (1999). Molecular classification of cancer: class discovery and class prediction by gene expression monitoring. *Science* 286 531–537. 10.1126/science.286.5439.531 10521349

[B25] Ha M. J. Baladandayuthapani V. Do K.-A. (2015). DINGO: differential network analysis in genomics. *Bioinformatics* 31 3413–3420. 10.1093/bioinformatics/btv406 26148744 PMC4751246

[B26] Huang D. W. Sherman B. T. Lempicki R. A. (2009). Bioinformatics enrichment tools: paths toward the comprehensive functional analysis of large gene lists. *Nucleic Acids Res.* 37 1–13. 10.1093/nar/gkn923 19033363 PMC2615629

[B27] Huang H.-C. Niu Y. Qin L.-X. (2015). Differential expression analysis for RNA-Seq: an overview of statistical methods and computational software: supplementary issue: sequencing platform modeling and analysis. *Cancer Inform.* 14:S21631. 10.4137/CIN.S21631 PMC4678998 26688660

[B28] Jardim V. C. Santos S. D. S. Fujita A. Buckeridge M. S. (2019). BioNetStat: a tool for biological networks differential analysis. *Front. Genet.* 10:594. 10.3389/fgene.2019.00594 PMC6598498 31293621

[B29] Jin W. Wu K. Li Y. Z. Yang W. T. Zou B. Zhang F. (2013). AML1-ETO targets and suppresses cathepsin G, a serine protease, which is able to degrade AML1-ETO in t(8;21) acute myeloid leukemia. *Oncogene* 32 1978–1987. 10.1038/onc.2012.204 22641217

[B30] Kakati T. Bhattacharyya D. K. Barah P. Kalita J. K. (2019). Comparison of methods for differential co-expression analysis for disease biomarker prediction. *Comp. Biol. Med.* 113:103380. 10.1016/j.compbiomed.2019.103380 31415946

[B31] Kanehisa M. Goto S. (2000). KEGG: kyoto encyclopedia of genes and genomes. *Nucleic Acids Res.* 28 27–30. 10592173 10.1093/nar/28.1.27 PMC102409

[B32] Kayano M. Shiga M. Mamitsuka H. (2014). Detecting differentially coexpressed genes from labeled expression data: a brief review. *IEEE/ACM Trans. Comput. Biol. Bioinform.* 11 154–167. 10.1109/tcbb.2013.2297921 26355515

[B33] Khan M. Carmona S. Sukhumalchandra P. Roszik J. Philips A. Perakis A. A. (2017). Cathepsin G is expressed by acute lymphoblastic leukemia and is a potential immunotherapeutic target. *Front. Immunol.* 8:1975. 10.3389/fimmu.2017.01975 PMC5790053 29422892

[B34] Kuleshov M. V. Jones M. R. Rouillard A. D. Fernandez N. F. Duan Q. Wang Z. (2016). Enrichr: a comprehensive gene set enrichment analysis web server 2016 update. *Nucleic Acids Res.* 44 W90–W97. 27141961 10.1093/nar/gkw377 PMC4987924

[B35] Kumari S. Nie J. Chen H.-S. Ma H. Stewart R. Li X. (2012). Evaluation of gene association methods for coexpression network construction and biological knowledge discovery. *PLoS One* 7:e50411. 10.1371/journal.pone.0050411 23226279 PMC3511551

[B36] Langfelder P. Horvath S. (2008). WGCNA: an R package for weighted correlation network analysis. *BMC Bioinform.* 9:559. 10.1186/1471-2105-9-559 PMC2631488 19114008

[B37] Langfelder P. Luo R. Oldham M. C. Horvath S. (2011). Is my network module preserved and reproducible? *PLoS Comput. Biol.* 7:e1001057. 10.1371/journal.pcbi.1001057 21283776 PMC3024255

[B38] Langfelder P. Zhang B. Horvath S. (2008). Defining clusters from a hierarchical cluster tree: the dynamic tree cut package for R. *Bioinformatics* 24 719–720. 10.1093/bioinformatics/btm563 18024473

[B39] Lichtblau Y. Zimmermann K. Haldemann B. Lenze D. Hummel M. Leser U. (2017). Comparative assessment of differential network analysis methods. *Brief. Bioinform.* 18 837–850. 27473063 10.1093/bib/bbw061

[B40] Liu B.-H. Yu H. Tu K. Li C. Li Y.-X. Li Y.-Y. (2010). DCGL: an R package for identifying differentially coexpressed genes and links from gene expression microarray data. *Bioinformatics* 26 2637–2638. 10.1093/bioinformatics/btq471 20801914 PMC2951087

[B41] McKenzie A. T. Katsyv I. Song W.-M. Wang M. Zhang B. (2016). DGCA: a comprehensive R package for differential gene correlation analysis. *BMC Syst. Biol.* 10:106. 10.1186/s12918-016-0349-1 PMC5111277 27846853

[B42] Petereit J. Smith S. Harris F. C. Schlauch K. A. (2016). petal: co-expression network modelling in R. *BMC Syst. Biol.* 10:51. 10.1186/s12918-016-0298-8 PMC4977474 27490697

[B43] Pollard K. S. Dudoit S. Van Der Laan M. J. (2005). “Multiple testing procedures: the multtest package and applications to genomics,” in *Bioinformatics and Computational Biology Solutions Using R and Bioconductor*, eds Gentleman R. Carey V. J. Huber W. Irizarry R. A. Dudoit S. (New York, NY: Springer), 249–271. 10.1007/0-387-29362-0_15

[B44] R Core Team (2018). R: A Language and Environment for Statistical Computing. R Foundation for Statistical Computing. Vienna, Austria.

[B45] Rahmatallah Y. Emmert-Streib F. Glazko G. (2014). Gene Sets Net Correlations Analysis (GSNCA): a multivariate differential coexpression test for gene sets. *Bioinformatics* 30 360–368. 10.1093/bioinformatics/btt687 24292935 PMC4023302

[B46] Ramanan V. K. Shen L. Moore J. H. Saykin A. J. (2012). Pathway analysis of genomic data: concepts, methods, and prospects for future development. *Trends Genet.* 28 323–332. 10.1016/j.tig.2012.03.004 22480918 PMC3378813

[B47] Ravasz E. Somera A. L. Mongru D. A. Oltvai Z. N. Barabási A.-L. (2002). Hierarchical organization of modularity in metabolic networks. *Science* 297 1551–1555. 10.1126/science.1073374 12202830

[B48] Ruan D. Young A. Montana G. (2015). Differential analysis of biological networks. *BMC Bioinform.* 16:327. 10.1186/s12859-015-0735-5 PMC4600256 26453322

[B49] Schott J. R. (2007). A test for the equality of covariance matrices when the dimension is large relative to the sample sizes. *Comput. Stat. Data Anal.* 51 6535–6542. 10.1016/j.csda.2007.03.004

[B50] Scrucca L. Fop M. Murphy T. B. Raftery A. E. (2016). mclust 5: clustering, classification and density estimation using Gaussian finite mixture models. *R J.* 8:289. 10.32614/rj-2016-021 PMC5096736 27818791

[B51] Shannon P. Markiel A. Ozier O. Baliga N. S. Wang J. T. Ramage D. (2003). Cytoscape: a software environment for integrated models of biomolecular interaction networks. *Genome Res.* 13 2498–2504. 10.1101/gr.1239303 14597658 PMC403769

[B52] Shi W. J. Zhuang Y. Russell P. H. Hobbs B. D. Parker M. M. Castaldi P. J. (2019). Unsupervised discovery of phenotype-specific multi-omics networks. *Bioinformatics* 35 4336–4343. 10.1093/bioinformatics/btz226 30957844 PMC6931269

[B53] Shojaie A. (2020). Differential network analysis: a statistical perspective. *Wiley Interdiscip. Rev. Comput. Stat.* 13:e1508. 10.1002/wics.1508 PMC10088462 37050915

[B54] Singh A. J. Ramsey S. A. Filtz T. M. Kioussi C. (2018). Differential gene regulatory networks in development and disease. *Cell. Mol. Life Sci.* 75 1013–1025. 10.1007/s00018-017-2679-6 29018868 PMC11105524

[B55] Siska C. Bowler R. Kechris K. (2016). The discordant method: a novel approach for differential correlation. *Bioinformatics* 32 690–696. 10.1093/bioinformatics/btv633 26520855 PMC5006287

[B56] Siska C. Kechris K. (2017). Differential correlation for sequencing data. *BMC Res. Notes* 10:54. 10.1186/s13104-016-2331-9 PMC5244536 28103954

[B57] Soneson C. Delorenzi M. (2013). A comparison of methods for differential expression analysis of RNA-seq data. *BMC Bioinform.* 14:91. 10.1186/1471-2105-14-91 PMC3608160 23497356

[B58] Tesson B. M. Breitling R. Jansen R. C. (2010). DiffCoEx: a simple and sensitive method to find differentially coexpressed gene modules. *BMC Bioinform.* 11:497. 10.1186/1471-2105-11-497 20925918 PMC2976757

[B59] Tibshirani R. Hastie T. Narasimhan B. Chu G. (2003). Class prediction by nearest shrunken centroids, with applications to DNA microarrays. *Stat. Sci.* 18 104–117.

[B60] van Dam S. Vosa U. Van Der Graaf A. Franke L. De Magalhaes J. P. (2018). Gene co-expression analysis for functional classification and gene–disease predictions. *Brief. Bioinform.* 19 575–592. 28077403 10.1093/bib/bbw139 PMC6054162

[B61] Wang T. Ren Z. Ding Y. Fang Z. Sun Z. Macdonald M. L. (2016). FastGGM: an efficient algorithm for the inference of gaussian graphical model in biological networks. *PLoS Comput. Biol.* 12:e1004755. 10.1371/journal.pcbi.1004755 26872036 PMC4752261

[B62] Watson M. (2006). CoXpress: differential co-expression in gene expression data. *BMC Bioinform.* 7:509. 10.1186/1471-2105-7-509 PMC1660556 17116249

[B63] Xu R. Wunsch D. C. (2010). Clustering algorithms in biomedical research: a review. *IEEE Rev. Biomed. Eng.* 3 120–154. 10.1109/rbme.2010.2083647 22275205

[B64] Zhang B. Horvath S. (2005). A general framework for weighted gene co-expression network analysis. *Stat. Appl. Genet. Mol. Biol.* 4:17. 10.2202/1544-6115.1128 16646834

[B65] Zhang R. Ren Z. Chen W. (2018). SILGGM: an extensive R package for efficient statistical inference in large-scale gene networks. *PLoS Comput. Biol.* 14:e1006369. 10.1371/journal.pcbi.1006369 30102702 PMC6107288

