# Supplementary material for: Comparing Statistical Tests for Differential Network Analysis of Gene Modules
Source: Front Genet. 2021 May 19;12:630215. doi: 10.3389/fgene.2021.630215 (PMC8170128; doi:10.3389/fgene.2021.630215)
Supplement: Supplementary file 1 [file Data_Sheet_1.docx]

Supplementary Material

# Supplementary Data

## Supplementary Tables

## Supplementary Figures

**Supplementary Table 1**: Summary of simulation settings

|  | **N** | $\boldsymbol{\rho}$ | **P** | $\boldsymbol{\gamma}$ | **#perm** | **#Modules** | **Tables, Figures** |
| --- | --- | --- | --- | --- | --- | --- | --- |
| **Null Simulations** |  |  |  |  |  |  |  |
| CS | 25 | 0.3, 0.7 | 10, 50, 100 |  | 3000 | 1000 | Table 1 |
| AR1 | 25 | 0.3, 0.7 | 10, 50, 100 |  | 3000 | 1000 | Table 2 |
| Hub | 50, 100 | 0.7 | 10, 50 |  | 3000 | 1000 | Table 3 |
| **DCM Simulations** |  |  |  |  |  |  |  |
| CS: correlations dropped to 0 | 25 | 0.3, 0.7 | 10, 50, 100 | 0.1, 0.4, 0.7 | 3000 | 1000 | Figure 1, STable 2 |
| AR1: correlations dropped to 0 | 25 | 0.7 |  | 0.1, 0.4, 0.7 | 3000 | 1000 | Figure 2, STable 3 |
| CS: half of changed corrs. increase 50%, half decrease 50% | 25 | 0.5 | 10, 50, 100 | 0.1, 0.4, 0.7 | 3000 | 1000 | Figure 3, STable 4 |
| CS: change sign | 25 | 0.5 | 10, 50, 100 | 0.1, 0.4, 0.7 | 3000 | 1000 | SFigure 1  STable 5 |
| Hub: correlations with hub gene dropped to 0 | 50, 100 | 0.7 | 10, 50 | 0.1, 0.4, 0.7 | 3000 | 1000 | Figure 4, STable 6 |
| **DCM Sims: Comparing TOM vs Correlation** |  |  |  |  | 2000 | 100 | SFigure 2 |

- STable: Supplementary Table; SFigure: Supplementary Figure
- N : # samples per group (there are 2 groups, e.g. cases vs controls)
- $\rho$ : main correlation parameter for CS, AR1 and hub gene correlation structures, see main manuscript Section 2.3.1 for details
- $P$: # genes within the module
- $\gamma$ : the proportion of correlations that are changed between the two groups
- #perm : number of permutations used to calculate p-values
- #Modules: the total number of simulated modules, i.e. simulation replicates

**Supplementary Table 2**: True positive rates of methods for DCM simulations “CS With Correlations Dropped to Zero”

| $\rho$ | $P$ | $\gamma$ | PND4 | PND6 | PND8 | PND20 | DI | MAD | pairedT | wilcoxSRT | GSNCA | GHD | QAP | GCOR |
| --- | --- | --- | --- | --- | --- | --- | --- | --- | --- | --- | --- | --- | --- | --- |
| 0.3 | 10 | 0.1 | **0.10** | 0.09 | 0.10 | 0.10 | **0.10** | 0.09 | 0.05 | 0.07 | 0.05 | **0.10** | 0.03 | 0.06 |
| 0.3 | 10 | 0.4 | **0.32** | 0.28 | 0.26 | 0.21 | **0.32** | **0.31** | 0.11 | 0.21 | 0.04 | 0.25 | 0.01 | 0.09 |
| 0.3 | 10 | 0.7 | 0.50 | 0.43 | 0.38 | 0.30 | **0.57** | **0.56** | 0.40 | **0.55** | 0.05 | 0.22 | 0.00 | 0.07 |
| 0.3 | 50 | 0.1 | **0.15** | **0.15** | **0.15** | 0.12 | 0.13 | 0.12 | 0.07 | 0.12 | 0.04 | **0.24** | 0.02 | 0.04 |
| 0.3 | 50 | 0.4 | **0.66** | **0.64** | 0.59 | 0.37 | **0.63** | 0.62 | 0.40 | 0.52 | 0.02 | 0.54 | 0.00 | 0.03 |
| 0.3 | 50 | 0.7 | **0.96** | 0.94 | 0.91 | 0.53 | **0.97** | **0.96** | 0.86 | 0.93 | 0.04 | 0.59 | 0.00 | 0.02 |
| 0.3 | 100 | 0.1 | 0.15 | **0.17** | **0.18** | 0.15 | 0.13 | 0.12 | 0.09 | 0.15 | 0.03 | **0.25** | 0.02 | 0.05 |
| 0.3 | 100 | 0.4 | **0.72** | **0.72** | **0.71** | 0.44 | 0.70 | 0.69 | 0.57 | 0.68 | 0.02 | 0.46 | 0.00 | 0.01 |
| 0.3 | 100 | 0.7 | **0.98** | 0.97 | 0.96 | 0.70 | **0.98** | **0.99** | 0.96 | 0.98 | 0.04 | 0.48 | 0.00 | 0.01 |
| 0.7 | 10 | 0.1 | 0.52 | 0.62 | **0.65** | **0.66** | 0.32 | 0.20 | 0.02 | 0.13 | 0.23 | **0.73** | 0.02 | 0.03 |
| 0.7 | 10 | 0.4 | **0.92** | **0.91** | **0.91** | 0.88 | 0.90 | 0.87 | 0.41 | 0.64 | 0.28 | 0.89 | 0.00 | 0.01 |
| 0.7 | 10 | 0.7 | **0.99** | 0.99 | 0.99 | 0.96 | **1.00** | **1.00** | 0.88 | 0.95 | 0.24 | 0.90 | 0.00 | 0.00 |
| 0.7 | 50 | 0.1 | 0.74 | **0.77** | **0.78** | **0.77** | 0.69 | 0.66 | 0.61 | 0.75 | 0.26 | 0.41 | 0.16 | 0.00 |
| 0.7 | 50 | 0.4 | **0.99** | **0.99** | 0.99 | 0.98 | **0.99** | **0.99** | **0.99** | **1.00** | 0.07 | 0.31 | 0.03 | 0.00 |
| 0.7 | 50 | 0.7 | **1.00** | **1.00** | **1.00** | 1.00 | **1.00** | **1.00** | **1.00** | **1.00** | 0.01 | 0.43 | 0.00 | 0.00 |
| 0.7 | 100 | 0.1 | 0.87 | **0.88** | 0.87 | 0.84 | 0.86 | 0.86 | **0.89** | **0.93** | 0.25 | 0.08 | 0.25 | 0.00 |
| 0.7 | 100 | 0.4 | **1.00** | **1.00** | **1.00** | 0.99 | **1.00** | **1.00** | **1.00** | **1.00** | 0.03 | 0.01 | 0.06 | 0.00 |
| 0.7 | 100 | 0.7 | **1.00** | **1.00** | **1.00** | **1.00** | **1.00** | **1.00** | **1.00** | **1.00** | 0.00 | 0.00 | 0.00 | 0.00 |

-All settings use N=25 subjects per group
-$\rho$ : compound symmetric correlation parameter; $P$ : # of genes in the module; $\gamma$ : proportion of correlations dropped to zero
-True positive rates in **bold** are in the top three highest of that row (ties are given the same rank)

Supplementary Table 3: True positive rates of methods for DCM simulations “AR1 With Correlations Dropped to Zero”

| $\rho$ | $P$ | $\gamma$ | PND4 | PND6 | PND8 | PND20 | DI | MAD | pairedT | wilcoxSRT | GSNCA | GHD | QAP | GCOR |
| --- | --- | --- | --- | --- | --- | --- | --- | --- | --- | --- | --- | --- | --- | --- |
| 0.7 | 10 | 0.1 | 0.14 | **0.19** | **0.21** | **0.23** | 0.09 | 0.08 | 0.03 | 0.06 | 0.06 | 0.14 | 0.03 | 0.02 |
| 0.7 | 10 | 0.4 | **0.60** | **0.60** | **0.60** | 0.57 | 0.50 | 0.38 | 0.09 | 0.18 | 0.07 | 0.52 | 0.01 | 0.00 |
| 0.7 | 10 | 0.7 | **0.90** | **0.87** | 0.83 | 0.75 | **0.89** | 0.84 | 0.30 | 0.46 | 0.08 | 0.70 | 0.00 | 0.01 |
| 0.7 | 50 | 0.1 | 0.12 | **0.15** | **0.17** | **0.17** | 0.10 | 0.10 | 0.06 | 0.08 | 0.06 | 0.10 | 0.02 | 0.02 |
| 0.7 | 50 | 0.4 | **0.71** | **0.74** | **0.72** | 0.50 | 0.55 | 0.44 | 0.13 | 0.20 | 0.06 | 0.51 | 0.00 | 0.00 |
| 0.7 | 50 | 0.7 | **0.99** | **0.99** | **0.98** | 0.78 | 0.97 | 0.91 | 0.42 | 0.50 | 0.10 | 0.91 | 0.00 | 0.00 |
| 0.7 | 100 | 0.1 | 0.11 | **0.15** | **0.16** | **0.15** | 0.10 | 0.09 | 0.04 | 0.05 | 0.06 | 0.10 | 0.02 | 0.02 |
| 0.7 | 100 | 0.4 | **0.73** | **0.80** | **0.81** | 0.49 | 0.54 | 0.41 | 0.14 | 0.20 | 0.08 | 0.51 | 0.00 | 0.00 |
| 0.7 | 100 | 0.7 | **1.00** | **1.00** | **1.00** | 0.77 | 0.98 | 0.92 | 0.44 | 0.48 | 0.16 | 0.96 | 0.00 | 0.00 |

-All settings use N=25 subjects per group
-$\rho$ : AR1 correlation parameter; $P$ : number of genes in the module; $\gamma$ : proportion of correlations dropped to zero
-True positive rates in **bold** are in the top three highest of that row (ties are given the same rank)

Supplementary Table 4: True positive rates of methods for DCM simulations “CS Where Half of the Changed Correlations Increase 50%, Half Decrease 50%”

| $\rho$ | $P$ | $\gamma$ | PND4 | PND6 | PND8 | PND20 | DI | MAD | pairedT | wilcoxSRT | GSNCA | GHD | QAP | GCOR |
| --- | --- | --- | --- | --- | --- | --- | --- | --- | --- | --- | --- | --- | --- | --- |
| 0.5 | 10 | 0.1 | 0.07 | 0.08 | **0.09** | **0.09** | 0.06 | 0.05 | 0.03 | 0.03 | 0.07 | **0.12** | 0.04 | 0.07 |
| 0.5 | 10 | 0.4 | 0.15 | **0.16** | **0.16** | 0.15 | 0.13 | 0.11 | 0.02 | 0.03 | 0.10 | **0.38** | 0.02 | 0.07 |
| 0.5 | 10 | 0.7 | **0.22** | **0.23** | **0.22** | 0.21 | 0.20 | 0.18 | 0.01 | 0.03 | 0.16 | **0.56** | 0.01 | 0.06 |
| 0.5 | 50 | 0.1 | 0.08 | 0.09 | **0.10** | **0.11** | 0.07 | 0.07 | 0.05 | 0.08 | 0.05 | **0.18** | 0.04 | 0.05 |
| 0.5 | 50 | 0.4 | 0.21 | **0.23** | **0.23** | 0.19 | 0.20 | 0.19 | 0.12 | 0.21 | 0.05 | **0.34** | 0.04 | 0.04 |
| 0.5 | 50 | 0.7 | **0.36** | **0.36** | 0.36 | 0.30 | 0.34 | 0.32 | 0.23 | 0.35 | 0.04 | **0.39** | 0.05 | 0.02 |
| 0.5 | 100 | 0.1 | 0.10 | 0.10 | 0.10 | **0.10** | 0.08 | 0.08 | 0.06 | **0.12** | 0.05 | **0.20** | 0.05 | 0.04 |
| 0.5 | 100 | 0.4 | 0.33 | **0.34** | **0.34** | 0.28 | 0.32 | 0.31 | 0.28 | **0.40** | 0.04 | 0.18 | 0.14 | 0.02 |
| 0.5 | 100 | 0.7 | **0.56** | **0.56** | 0.55 | 0.41 | 0.54 | 0.54 | 0.53 | **0.64** | 0.04 | 0.13 | 0.16 | 0.02 |

-All settings use N=25 subjects per group
-$\rho$ : compound symmetric correlation parameter, $P$ : # of genes in module, $\gamma$ : proportion of correlations changed between groups
-True positive rates in **bold** are in the top three highest of that row (ties are given the same rank)

**Supplementary Table 5**: True positive rates of methods for DCM simulations “CS Where Correlations Change Direction” (from 0.5 to -0.5)

| $\rho$ | P | $\gamma$ | PND4 | PND6 | PND8 | PND20 | DI | MAD | pairedT | wilcoxSRT | GSNCA | GHD | QAP | GCOR |
| --- | --- | --- | --- | --- | --- | --- | --- | --- | --- | --- | --- | --- | --- | --- |
| 0.5 | 10 | 0.1 | 0.70 | **0.75** | **0.77** | **0.75** | 0.47 | 0.27 | 0.03 | 0.13 | 0.07 | 0.72 | 0.01 | 0.04 |
| 0.5 | 10 | 0.4 | **1.00** | **1.00** | 0.99 | 0.98 | **1.00** | **1.00** | 0.73 | 0.89 | 0.01 | 0.96 | 0.00 | 0.01 |
| 0.5 | 10 | 0.7 | **1.00** | **1.00** | **1.00** | **1.00** | **1.00** | **1.00** | 1.00 | **1.00** | 0.00 | 0.95 | 0.04 | 0.00 |
| 0.5 | 50 | 0.1 | **0.83** | **0.86** | **0.87** | 0.80 | 0.77 | 0.73 | 0.62 | 0.75 | 0.04 | 0.57 | 0.03 | 0.00 |
| 0.5 | 50 | 0.4 | **1.00** | **1.00** | **1.00** | **1.00** | **1.00** | **1.00** | **1.00** | **1.00** | 0.01 | 0.69 | 0.00 | 0.00 |
| 0.5 | 50 | 0.7 | **1.00** | **1.00** | **1.00** | 1.00 | **1.00** | **1.00** | **1.00** | **1.00** | 0.11 | 0.55 | 0.00 | 0.00 |
| 0.5 | 100 | 0.1 | 0.92 | **0.93** | **0.93** | 0.85 | 0.90 | 0.89 | 0.90 | **0.95** | 0.01 | 0.09 | 0.11 | 0.00 |
| 0.5 | 100 | 0.4 | **1.00** | **1.00** | **1.00** | 1.00 | **1.00** | **1.00** | **1.00** | **1.00** | 0.04 | 0.00 | 0.00 | 0.00 |
| 0.5 | 100 | 0.7 | **1.00** | **1.00** | **1.00** | **1.00** | **1.00** | **1.00** | **1.00** | **1.00** | 0.26 | 0.00 | 0.00 | 0.00 |

-All settings use N=25 subjects per group
-$\rho$ : compound symmetric correlation parameter; $P$ : # of genes in the module; $\gamma$ : proportion of correlations changed to -0.5
-True positive rates in **bold** are in the top three highest of that row (ties are given the same rank)

Supplementary Table 6: True positive rates of methods for the hub gene DCM simulations

| N | $\rho$ | $P$ | $\gamma$ | PND4 | PND6 | PND8 | PND20 | DI | MAD | pairedT | wilcoxSRT | GSNCA | GHD | QAP | GCOR |
| --- | --- | --- | --- | --- | --- | --- | --- | --- | --- | --- | --- | --- | --- | --- | --- |
| 50 | 0.7 | 10 | 0.1 | 0.38 | **0.52** | **0.58** | **0.62** | 0.13 | 0.08 | 0.03 | 0.06 | 0.14 | 0.31 | 0.03 | 0.00 |
| 50 | 0.7 | 10 | 0.4 | 0.95 | **0.96** | **0.96** | 0.95 | 0.76 | 0.38 | 0.02 | 0.11 | 0.37 | **0.97** | 0.00 | 0.16 |
| 50 | 0.7 | 10 | 0.7 | **1.00** | **1.00** | 0.99 | 0.98 | 0.97 | 0.70 | 0.02 | 0.10 | 0.49 | **1.00** | 0.00 | 0.64 |
| 100 | 0.7 | 50 | 0.1 | 0.18 | **0.49** | **0.70** | **0.83** | 0.07 | 0.06 | 0.04 | 0.06 | 0.19 | 0.39 | 0.03 | 0.00 |
| 100 | 0.7 | 50 | 0.4 | 0.95 | **0.99** | **1.00** | **1.00** | 0.27 | 0.12 | 0.03 | 0.07 | 0.32 | 0.99 | 0.00 | 0.31 |
| 100 | 0.7 | 50 | 0.7 | 1.00 | **1.00** | **1.00** | **1.00** | 0.75 | 0.23 | 0.02 | 0.09 | 0.47 | **1.00** | 0.00 | 0.90 |

-N: number of subjects per group
-$\rho$ : correlation of non hub genes with hub gene, $P$ : # of genes in the module, $\gamma$ : proportion of hub gene correlations dropped to zero
-True positive rates in **bold** are in the top three highest of that row (ties are given the same rank)

**Supplementary Table 7:** Comparing overall performance of tests across all 51 simulation scenarios

| Test | Within Top 3 best TPRs:  N(%) | Within 5% of best TPR:  N(%) | Median TPR |
| --- | --- | --- | --- |
| PND4 | 30 (58.8%) | 33 (64.7%) | 0.73 |
| PND6 | 41 (80.4%) | 37 (72.5%) | 0.77 |
| PND8 | 36 (70.6%) | 34 (66.7%) | 0.78 |
| PND20 | 17 (33.3%) | 26 (51.0%) | 0.71 |
| DI | 18 (35.3%) | 25 (49.0%) | 0.63 |
| MAD | 15 (29.4%) | 19 (37.3%) | 0.54 |
| pairedT | 9 (17.6%) | 13 (25.5%) | 0.28 |
| wilcoxSRT | 15 (29.4%) | 20 (39.2%) | 0.40 |
| GSNCA | 0 (0.0%) | 2 (3.9%) | 0.06 |
| GHD | 14 (27.5%) | 20 (39.2%) | 0.41 |
| QAP | 0 (0.0%) | 0 (0.0%) | 0.01 |
| GCOR | 0 (0.0%) | 2 (3.9%) | 0.01 |

Supplementary Table 8 Summary of all modules from the Golub dataset. Module names with prefix “ALL” were derived in the ALL group, while modules with prefix “AML” were derived in the AML group. When testing whether the module is differentially co-expressed between the ALL and AML groups, p-values and FDR adjusted p-values are reported for a subset of the top performing tests from our simulations: PND6, DI, MAD, and GHD. P-values < 0.01 are in scientific notation (e = *10^).

|  |  | p-values | | | | FDR-adjusted p-values | | | |
| --- | --- | --- | --- | --- | --- | --- | --- | --- | --- |
| Module | N Genes | PND6 | DI | MAD | GHD | PND6 | DI | MAD | GHD |
| ALL_1 | 94 | 0.18 | 0.22 | 0.25 | 0.23 | 0.21 | 0.24 | 0.27 | 0.34 |
| ALL_2 | 79 | 0.04 | 0.06 | 0.07 | 0.04 | 0.06 | 0.08 | 0.08 | 0.1 |
| ALL_3 | 35 | 0.88 | 0.84 | 0.8 | 0.88 | 0.88 | 0.84 | 0.8 | 0.91 |
| ALL_4 | 78 | 0.02 | 0.02 | 0.03 | 0.02 | 0.04 | 0.04 | 0.05 | 0.08 |
| ALL_5 | 58 | 5.3e-3 | 0.01 | 0.02 | 3.5e-3 | 0.01 | 0.03 | 0.04 | 0.04 |
| ALL_6 | 12 | 0.49 | 0.36 | 0.26 | 0.36 | 0.52 | 0.39 | 0.28 | 0.49 |
| ALL_7 | 101 | 3.1e-3 | 6.8e-3 | 0.01 | 0.04 | 0.01 | 0.02 | 0.02 | 0.11 |
| ALL_8 | 140 | 0.04 | 0.05 | 0.06 | 0.06 | 0.06 | 0.07 | 0.08 | 0.14 |
| ALL_9 | 77 | 0.02 | 0.01 | 0.01 | 0.15 | 0.03 | 0.03 | 0.02 | 0.25 |
| ALL_10 | 95 | 0.02 | 0.02 | 0.02 | 0.02 | 0.04 | 0.04 | 0.04 | 0.08 |
| ALL_11 | 35 | 0.01 | 0.02 | 0.02 | 0.03 | 0.03 | 0.03 | 0.04 | 0.09 |
| ALL_12 | 61 | 0.07 | 0.05 | 0.05 | 0.01 | 0.09 | 0.07 | 0.07 | 0.07 |
| ALL_13 | 75 | 0.08 | 0.04 | 0.05 | 0.14 | 0.1 | 0.06 | 0.07 | 0.25 |
| ALL_14 | 115 | 0.04 | 0.05 | 0.05 | 0.02 | 0.06 | 0.07 | 0.07 | 0.08 |
| ALL_15 | 39 | 2.4e-3 | 1.3e-3 | 1.4e-3 | 0.08 | 9.3e-3 | 4.5e-3 | 4.6e-3 | 0.15 |
| ALL_16 | 80 | 1.0e-4 | 4.0e-4 | 9.0e-4 | 7.0e-4 | 8.6e-4 | 2.1e-3 | 3.7e-3 | 0.01 |
| ALL_17 | 74 | 0.62 | 0.58 | 0.54 | 0.38 | 0.64 | 0.6 | 0.56 | 0.5 |
| ALL_18 | 92 | 0.01 | 0.02 | 0.02 | 0.03 | 0.03 | 0.04 | 0.03 | 0.09 |
| ALL_19 | 37 | 7.0e-4 | 2.0e-4 | 3.0e-4 | 7.4e-3 | 4.3e-3 | 1.3e-3 | 1.8e-3 | 0.06 |
| ALL_20 | 37 | 0.23 | 0.08 | 0.04 | 0.06 | 0.26 | 0.1 | 0.06 | 0.14 |
| ALL_21 | 27 | 0.02 | 0.02 | 0.03 | 2.9e-3 | 0.03 | 0.04 | 0.05 | 0.04 |
| ALL_22 | 3 | 7.9e-3 | 7.9e-3 | 7.9e-3 | 6.9e-3 | 0.02 | 0.02 | 0.02 | 0.06 |
| ALL_23 | 74 | 3.7e-3 | 2.2e-3 | 1.9e-3 | 0.01 | 0.01 | 7.0e-3 | 5.8e-3 | 0.07 |
| ALL_24 | 59 | 2.5e-3 | 0.01 | 0.02 | 0.02 | 9.3e-3 | 0.02 | 0.03 | 0.08 |
| ALL_25 | 60 | 0.02 | 0.02 | 0.02 | 0.06 | 0.04 | 0.03 | 0.04 | 0.14 |
| ALL_26 | 75 | 0.01 | 0.01 | 0.02 | 0.07 | 0.03 | 0.03 | 0.03 | 0.15 |
| ALL_27 | 64 | 5.0e-4 | 5.0e-4 | 8.0e-4 | 7.9e-3 | 3.9e-3 | 2.3e-3 | 3.4e-3 | 0.06 |
| ALL_28 | 70 | 0.41 | 0.4 | 0.42 | 0.35 | 0.44 | 0.43 | 0.45 | 0.48 |
| ALL_29 | 57 | 0.27 | 0.25 | 0.29 | 0.19 | 0.29 | 0.28 | 0.31 | 0.29 |
| ALL_30 | 113 | 8.0e-4 | 1.0e-3 | 1.0e-3 | 5.9e-3 | 4.3e-3 | 3.9e-3 | 3.7e-3 | 0.06 |
| ALL_31 | 17 | 1.0e-4 | 1.0e-4 | 1.0e-4 | 8.0e-4 | 8.6e-4 | 7.8e-4 | 8.6e-4 | 0.01 |
| ALL_32 | 64 | 8.0e-4 | 2.6e-3 | 5.4e-3 | 5.9e-3 | 4.3e-3 | 7.7e-3 | 0.01 | 0.06 |
| ALL_33 | 107 | 0.09 | 0.07 | 0.07 | 0.04 | 0.12 | 0.08 | 0.08 | 0.11 |
| ALL_34 | 74 | 0.47 | 0.29 | 0.25 | 0.15 | 0.5 | 0.31 | 0.28 | 0.25 |
| ALL_35 | 75 | 0.14 | 0.08 | 0.06 | 0.08 | 0.16 | 0.1 | 0.08 | 0.15 |
| ALL_36 | 79 | 0.09 | 0.1 | 0.11 | 0.01 | 0.12 | 0.12 | 0.13 | 0.08 |
| ALL_37 | 31 | 0.13 | 0.14 | 0.18 | 0.08 | 0.16 | 0.16 | 0.2 | 0.15 |
| ALL_38 | 22 | 0.04 | 0.04 | 0.05 | 0.13 | 0.06 | 0.06 | 0.07 | 0.23 |
| ALL_39 | 79 | 1.0e-3 | 1.0e-3 | 1.1e-3 | 9.1e-3 | 4.5e-3 | 3.9e-3 | 3.9e-3 | 0.06 |
| ALL_40 | 116 | 1.0e-4 | 7.0e-4 | 1.0e-3 | 2.0e-4 | 8.6e-4 | 3.0e-3 | 3.7e-3 | 8.6e-3 |
| ALL_41 | 65 | 3.1e-3 | 2.3e-3 | 1.9e-3 | 0.18 | 0.01 | 7.1e-3 | 5.8e-3 | 0.28 |
| ALL_42 | 145 | 9.0e-4 | 1.3e-3 | 1.3e-3 | 5.0e-4 | 4.3e-3 | 4.5e-3 | 4.5e-3 | 0.01 |
| ALL_43 | 22 | 9.0e-4 | 1.0e-4 | 1.0e-4 | 0.02 | 4.3e-3 | 7.8e-4 | 8.6e-4 | 0.08 |
| ALL_44 | 16 | 0.02 | 4.5e-3 | 2.6e-3 | 0.41 | 0.04 | 0.01 | 7.5e-3 | 0.53 |
| ALL_45 | 56 | 1.7e-3 | 8.6e-3 | 0.01 | 0.02 | 7.0e-3 | 0.02 | 0.03 | 0.08 |
| ALL_46 | 47 | 7.0e-4 | 5.0e-4 | 5.0e-4 | 0.02 | 4.3e-3 | 2.3e-3 | 2.4e-3 | 0.08 |
| ALL_47 | 13 | 0.61 | 0.74 | 0.8 | 0.68 | 0.64 | 0.75 | 0.8 | 0.79 |
| ALL_48 | 4 | 0.1 | 0.08 | 0.07 | 0.17 | 0.13 | 0.1 | 0.09 | 0.28 |
| ALL_49 | 3 | 0.71 | 0.68 | 0.64 | 0.85 | 0.72 | 0.69 | 0.65 | 0.9 |
| AML_1 | 189 | 0.01 | 0.01 | 0.01 | 0.03 | 0.03 | 0.03 | 0.03 | 0.08 |
| AML_2 | 224 | 0.13 | 0.09 | 0.08 | 0.42 | 0.16 | 0.11 | 0.1 | 0.53 |
| AML_3 | 58 | 1.0e-4 | 1.0e-4 | 1.0e-4 | 0.15 | 8.6e-4 | 7.8e-4 | 8.6e-4 | 0.25 |
| AML_4 | 80 | 1.0e-4 | 1.0e-4 | 1.0e-4 | 0.99 | 8.6e-4 | 7.8e-4 | 8.6e-4 | 0.99 |
| AML_5 | 313 | 1.0e-4 | 1.0e-4 | 2.0e-4 | 2.0e-4 | 8.6e-4 | 7.8e-4 | 1.4e-3 | 8.6e-3 |
| AML_6 | 118 | 0.03 | 0.02 | 0.02 | 0.02 | 0.04 | 0.04 | 0.03 | 0.08 |
| AML_7 | 34 | 6.5e-3 | 5.0e-4 | 5.0e-4 | 0.09 | 0.02 | 2.3e-3 | 2.4e-3 | 0.16 |
| AML_8 | 158 | 0.07 | 0.06 | 0.06 | 0.07 | 0.1 | 0.08 | 0.08 | 0.15 |
| AML_9 | 272 | 0.17 | 0.19 | 0.19 | 0.34 | 0.2 | 0.21 | 0.22 | 0.48 |
| AML_10 | 13 | 1.0e-4 | 1.0e-4 | 1.0e-4 | 0.18 | 8.6e-4 | 7.8e-4 | 8.6e-4 | 0.28 |
| AML_11 | 265 | 0.03 | 0.03 | 0.04 | 0.03 | 0.04 | 0.05 | 0.06 | 0.09 |
| AML_12 | 133 | 2.7e-3 | 2.2e-3 | 2.3e-3 | 0.24 | 9.7e-3 | 7.0e-3 | 6.8e-3 | 0.36 |
| AML_13 | 16 | 0.02 | 2.8e-3 | 5.0e-4 | 0.75 | 0.03 | 8.0e-3 | 2.4e-3 | 0.85 |
| AML_14 | 146 | 1.0e-4 | 1.0e-4 | 1.0e-4 | 0.77 | 8.6e-4 | 7.8e-4 | 8.6e-4 | 0.86 |
| AML_15 | 37 | 7.0e-4 | 2.0e-4 | 2.0e-4 | 0.65 | 4.3e-3 | 1.3e-3 | 1.4e-3 | 0.77 |
| AML_16 | 189 | 0.02 | 0.02 | 0.02 | 0.02 | 0.04 | 0.04 | 0.04 | 0.08 |
| AML_17 | 17 | 0.01 | 0.02 | 0.02 | 0.06 | 0.03 | 0.04 | 0.04 | 0.14 |
| AML_18 | 14 | 0.04 | 6.6e-3 | 4.8e-3 | 0.34 | 0.06 | 0.02 | 0.01 | 0.48 |
| AML_19 | 28 | 1.6e-3 | 1.0e-4 | 1.0e-4 | 0.79 | 6.9e-3 | 7.8e-4 | 8.6e-4 | 0.86 |
| AML_20 | 72 | 1.0e-4 | 1.0e-4 | 1.0e-4 | 0.92 | 8.6e-4 | 7.8e-4 | 8.6e-4 | 0.94 |
| AML_21 | 9 | 0.04 | 0.03 | 0.03 | 0.34 | 0.06 | 0.05 | 0.05 | 0.48 |
| AML_22 | 20 | 3.2e-3 | 1.0e-4 | 1.0e-4 | 0.86 | 0.01 | 7.8e-4 | 8.6e-4 | 0.9 |
| AML_23 | 199 | 4.0e-3 | 4.2e-3 | 4.6e-3 | 0.08 | 0.01 | 0.01 | 0.01 | 0.15 |
| AML_24 | 94 | 0.07 | 0.06 | 0.05 | 0.94 | 0.1 | 0.08 | 0.07 | 0.95 |
| AML_25 | 21 | 0.09 | 0.03 | 0.02 | 0.06 | 0.11 | 0.05 | 0.03 | 0.14 |
| AML_26 | 61 | 0.05 | 0.03 | 0.03 | 0.02 | 0.08 | 0.05 | 0.05 | 0.08 |
| AML_27 | 17 | 7.1e-3 | 1.1e-3 | 7.0e-4 | 0.68 | 0.02 | 4.1e-3 | 3.2e-3 | 0.79 |
| AML_28 | 19 | 0.02 | 0.01 | 0.02 | 0.35 | 0.04 | 0.03 | 0.03 | 0.48 |
| AML_29 | 47 | 1.0e-4 | 4.0e-4 | 5.0e-4 | 0.84 | 8.6e-4 | 2.1e-3 | 2.4e-3 | 0.9 |
| AML_30 | 24 | 0.01 | 0.01 | 0.02 | 0.59 | 0.03 | 0.03 | 0.03 | 0.72 |
| AML_31 | 34 | 5.6e-3 | 3.6e-3 | 3.5e-3 | 0.02 | 0.01 | 1.0e-2 | 9.7e-3 | 0.08 |
| AML_32 | 22 | 5.3e-3 | 3.0e-4 | 3.0e-4 | 0.79 | 0.01 | 1.8e-3 | 1.8e-3 | 0.86 |
| AML_33 | 27 | 4.2e-3 | 1.0e-4 | 1.0e-4 | 0.46 | 0.01 | 7.8e-4 | 8.6e-4 | 0.56 |
| AML_34 | 6 | 0.14 | 0.11 | 0.1 | 0.43 | 0.16 | 0.13 | 0.12 | 0.53 |
| AML_35 | 22 | 0.14 | 0.11 | 0.1 | 0.73 | 0.16 | 0.13 | 0.12 | 0.84 |
| AML_36 | 33 | 0.67 | 0.52 | 0.51 | 0.39 | 0.69 | 0.55 | 0.54 | 0.51 |
| AML_37 | 20 | 0.08 | 0.07 | 0.1 | 0.1 | 0.11 | 0.09 | 0.12 | 0.18 |

**1.2 Supplementary Figures**

Supplementary Figure 1: True positive rates for the compound symmetric DCM simulations where a proportion of correlations change direction (0.5 to -0.5) between the two groups. Solid lines refer to the proposed PND tests, while dashed lines refer to pre-existing methods.


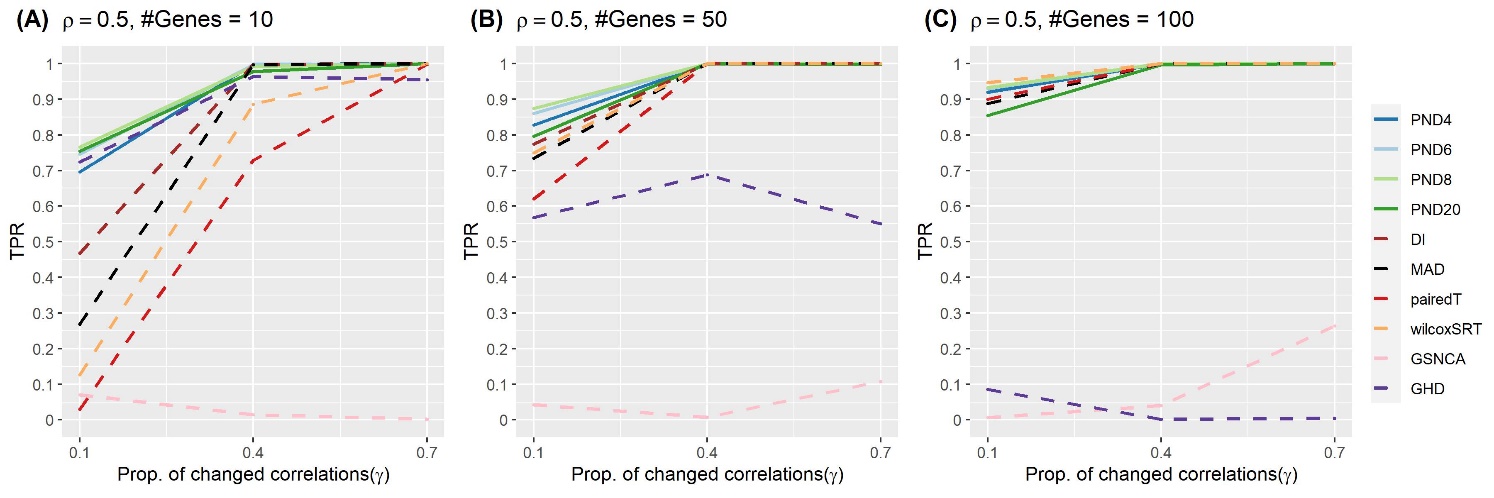


Supplementary Figure 2: comparing true positive rates between methods that use correlation (solid lines) and TOM (dashed lines)

**
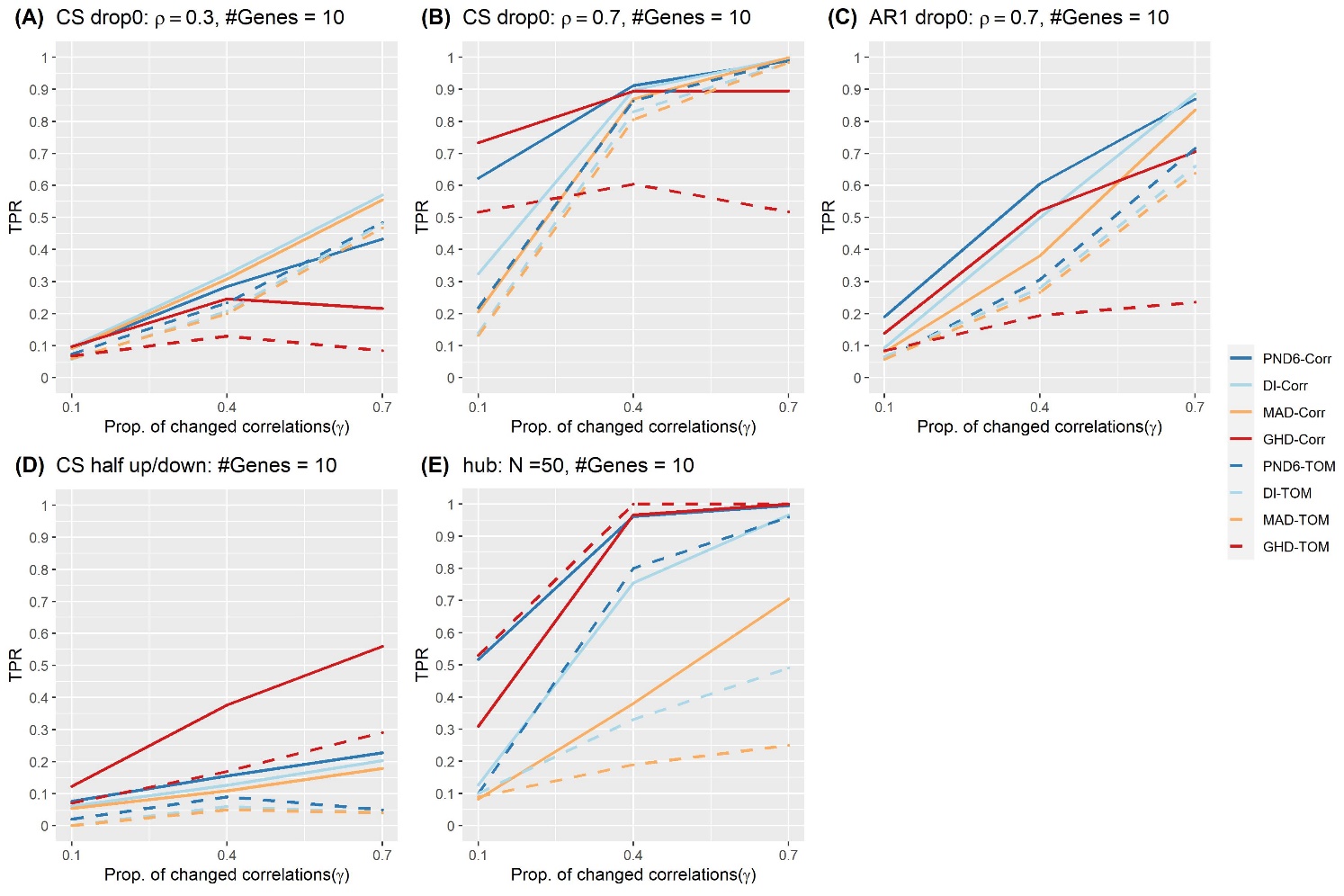
**

**Supplementary Figure 3.** Venn diagram comparing the total number of differentially co-expressed modules with FDR-adjusted p-values < 0.01


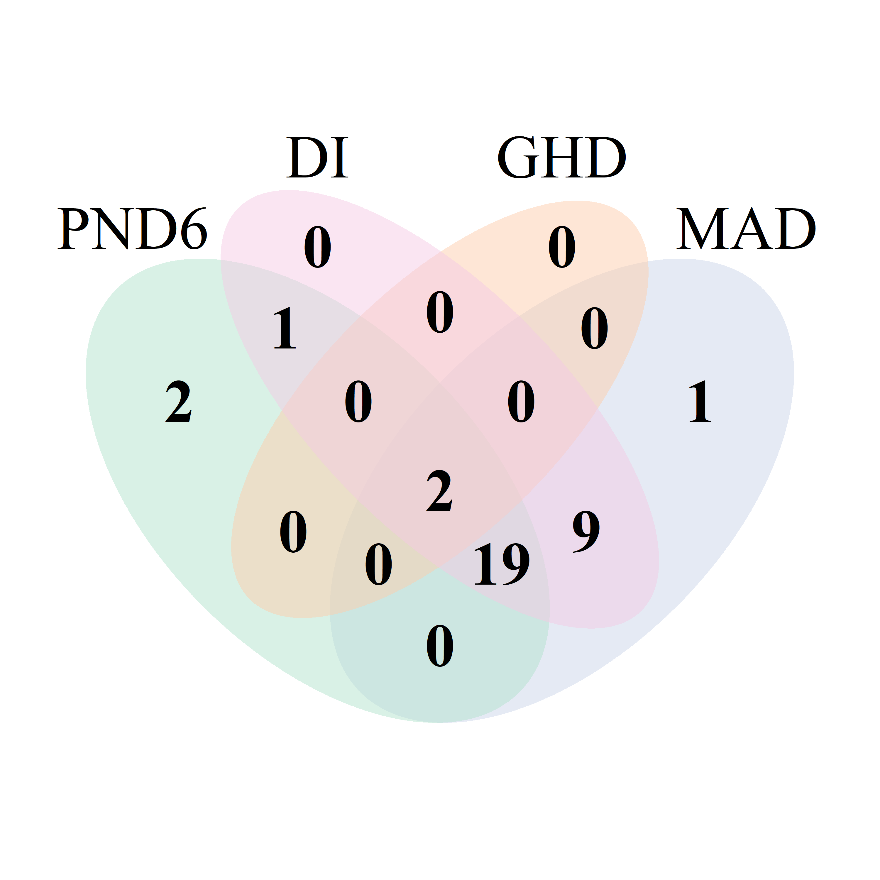


**Supplementary Figure 4.** Spearman’s correlation heatmaps for visualizing differential co-expression for several example modules that were derived in the ALL group.


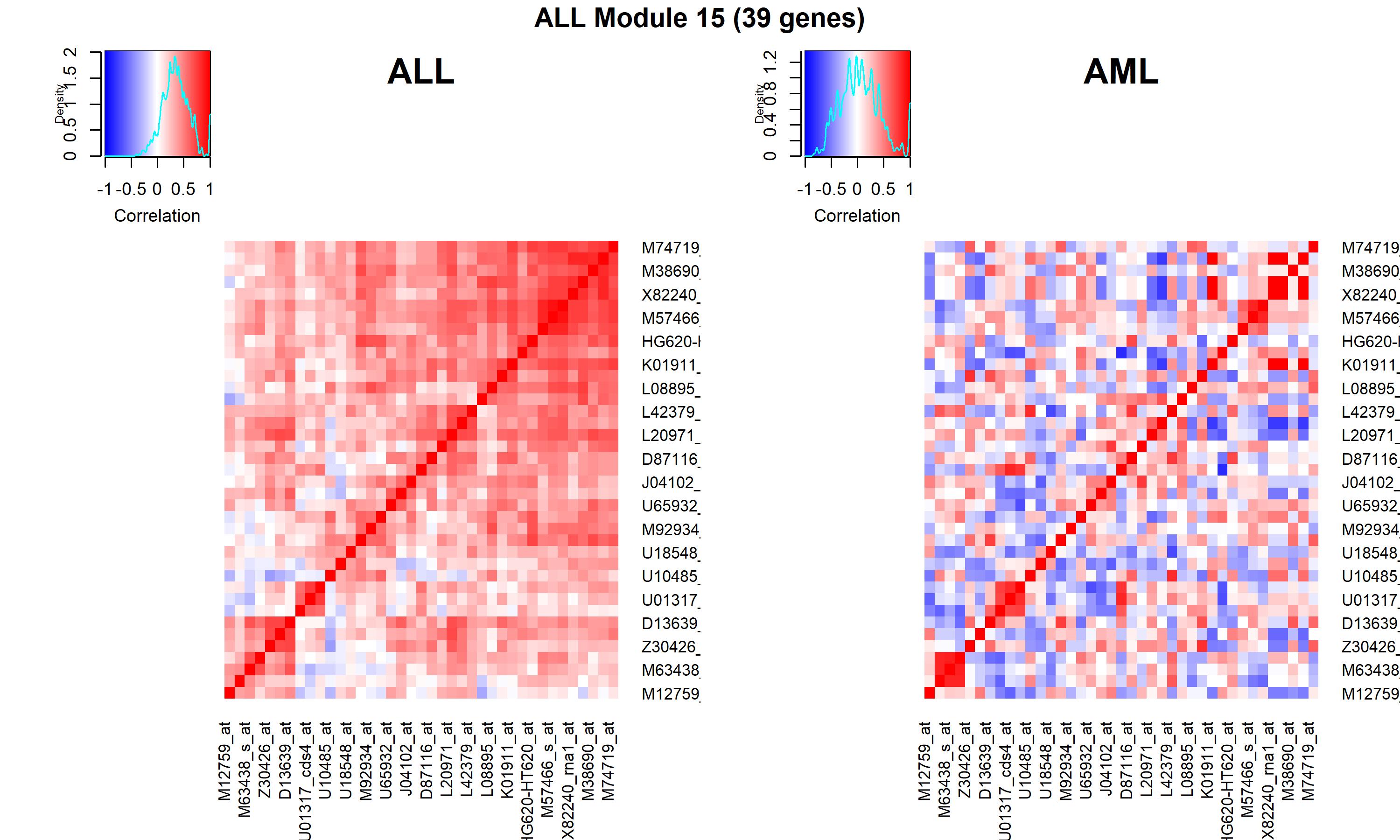


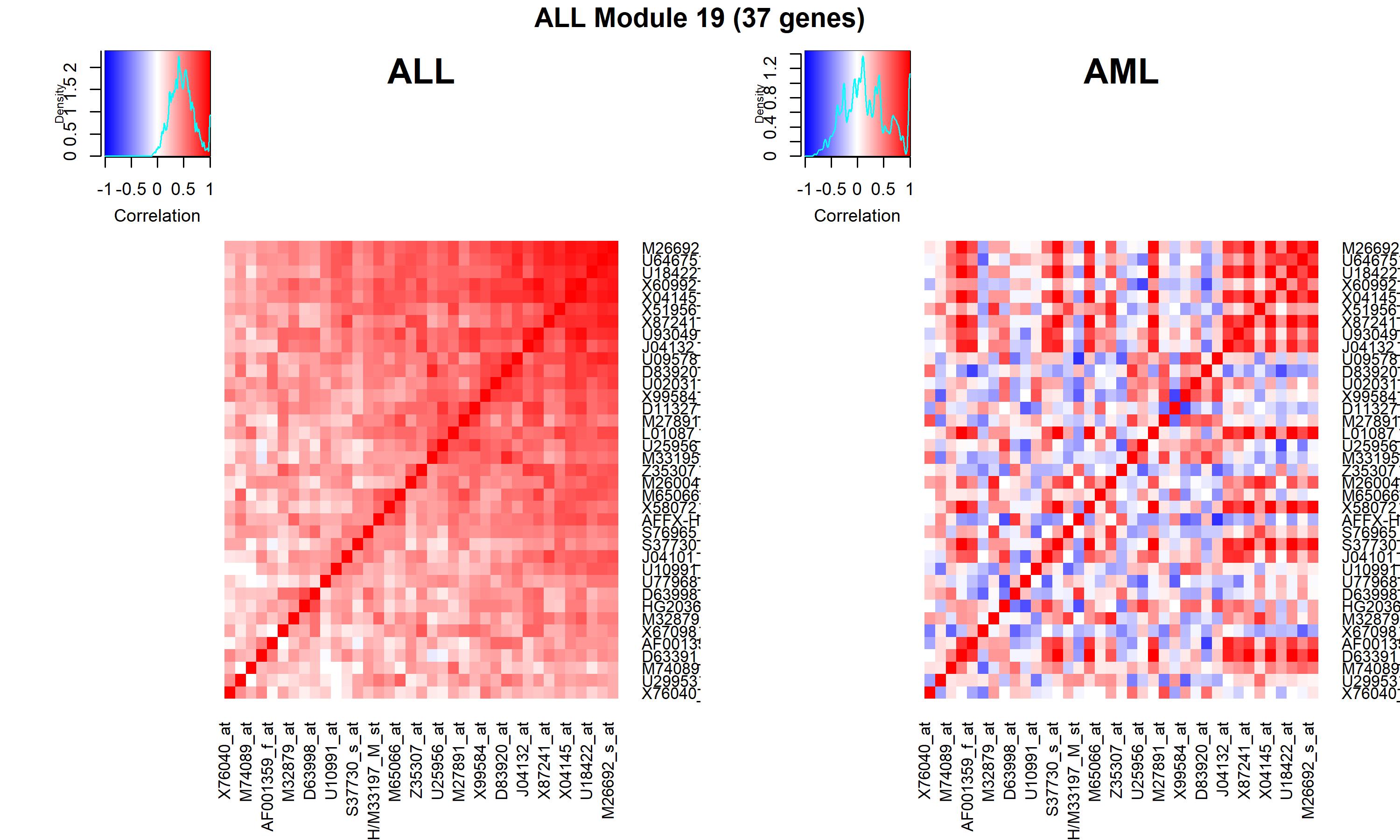


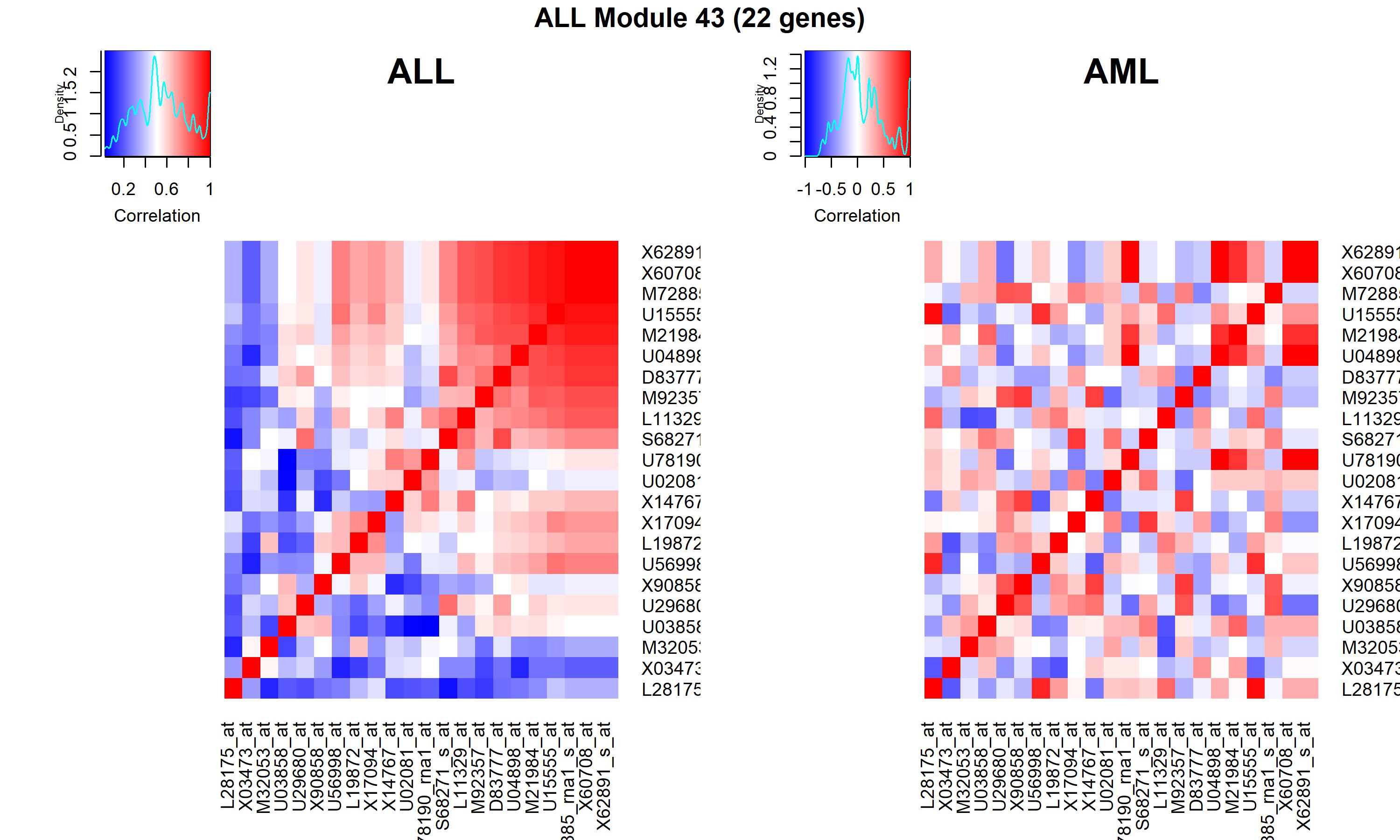


**Supplementary Figure 5.** Spearman’s correlation heatmaps for visualizing differential co-expression for several example modules that were derived in the AML group.


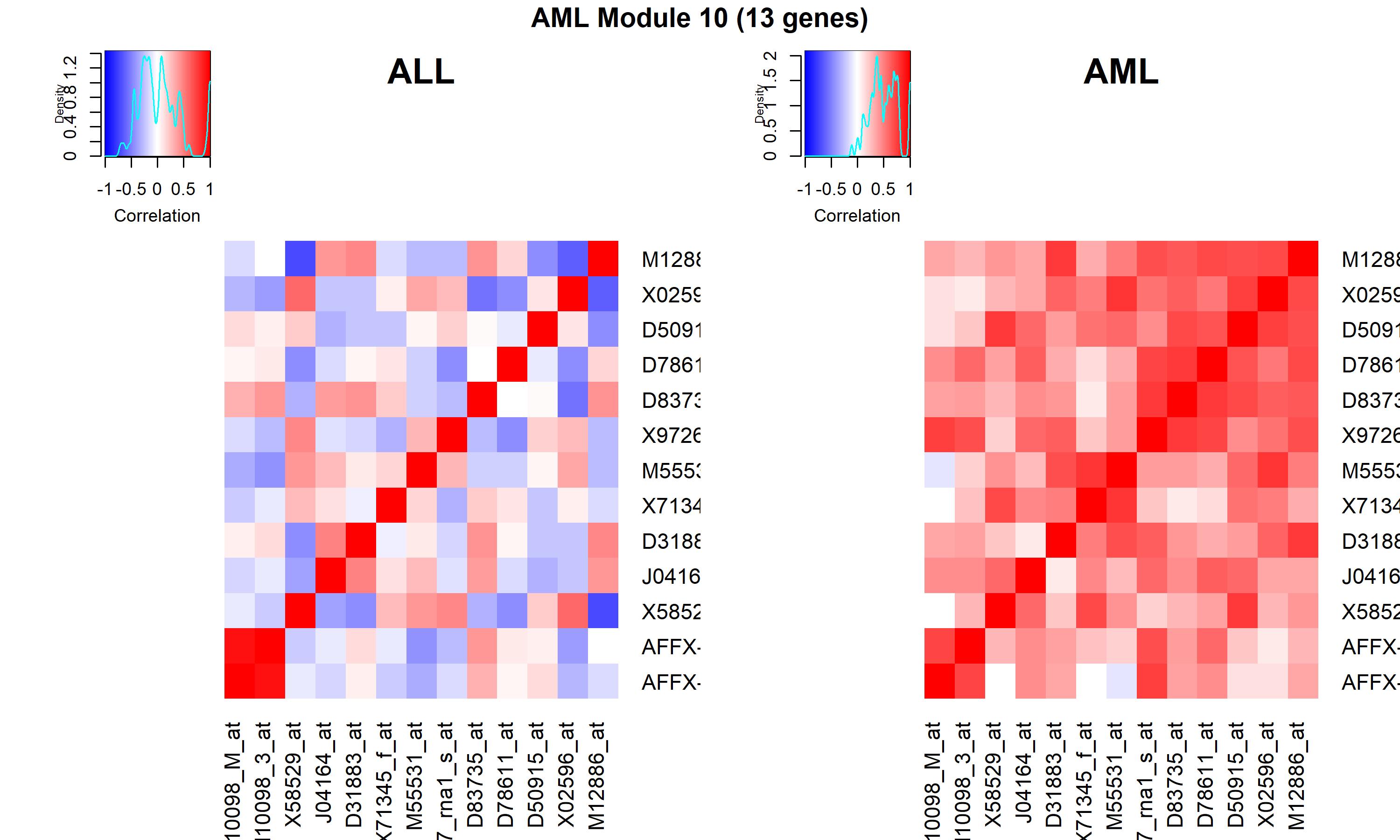


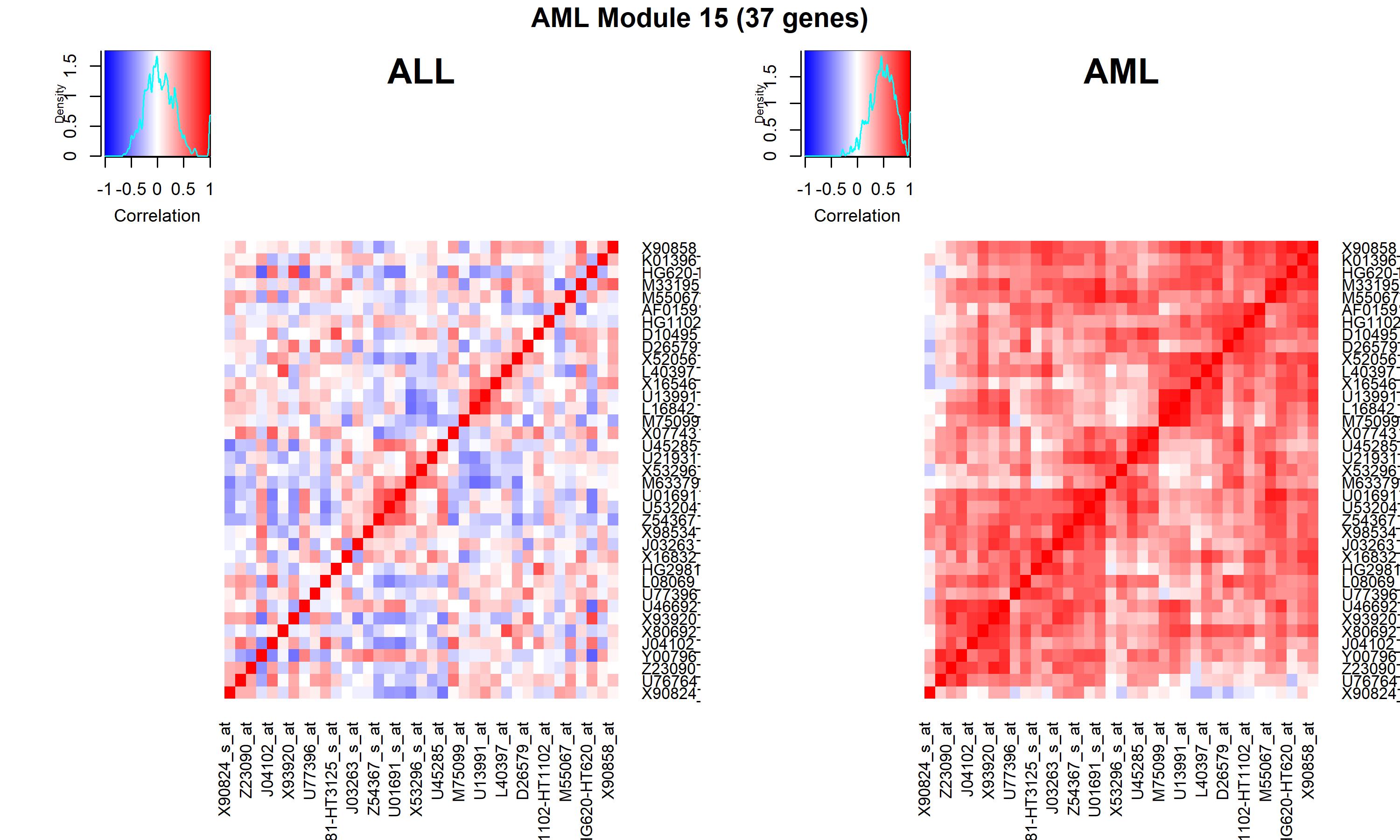


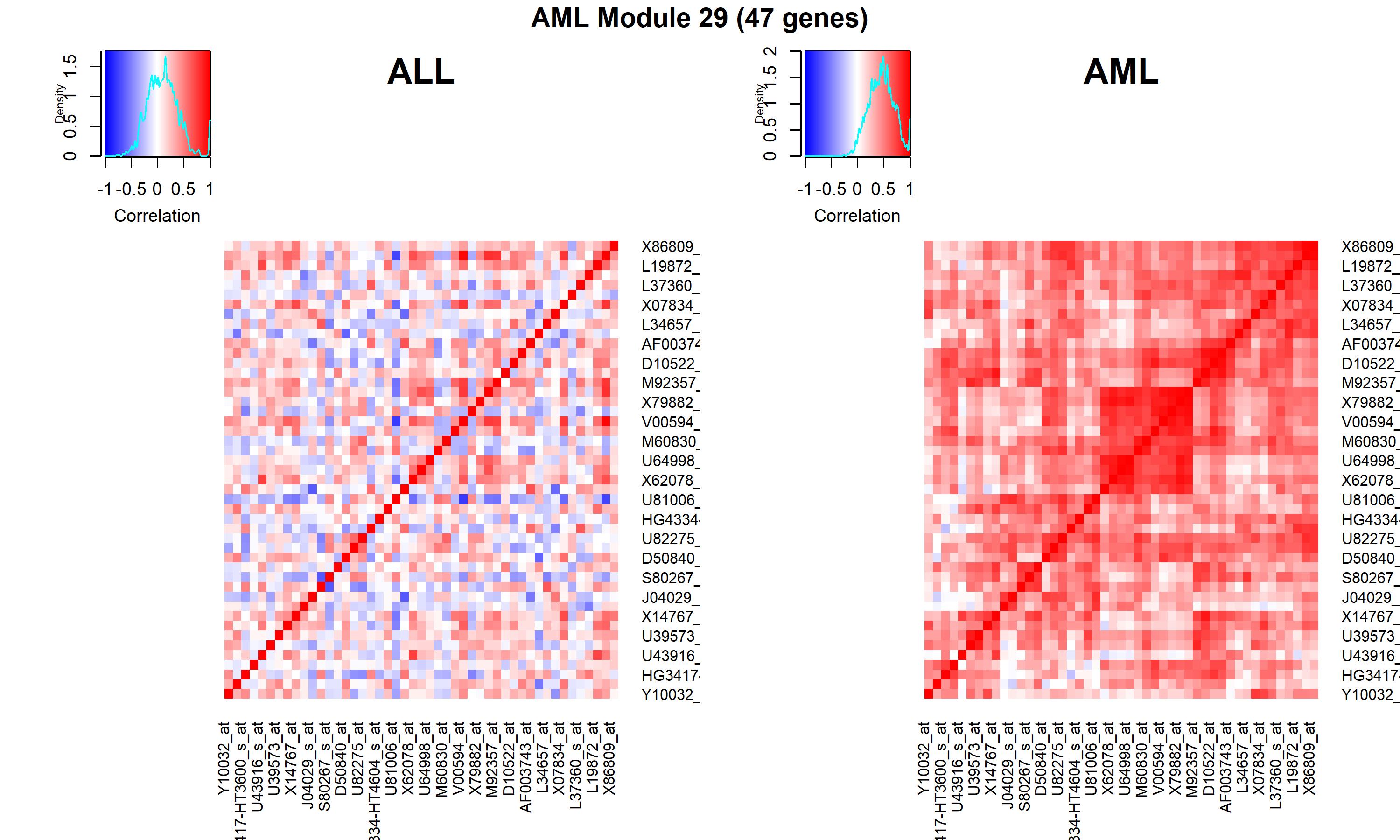


**Supplementary Figure 6.** PND6 Only differentially expressed co-expression module. The ALL_24 module originally identified in the ALL patient population was only differentially coexpressed using the PND6 method (FDR = 0.0093). For all networks, circles present individual probe sets. Labels are gene symbols for probe sets with annotation information in the Ensembl database. Otherwise, the original probe set identifier from Affymetrix was used. Red lines connecting circles indicate a positive correlation (correlation coefficient > 0.3) between the two probe sets. Blue lines connecting circles indicates a negative correlation between the two probe sets (correlation coefficient < -0.30). The intensity of the color and thickness of the lines are associated with the magnitude of the correlation between the two probe sets. **A) Co-expression patterns in the ALL population**. **B) Co-expression patterns in the AML population**. **C) Difference in correlation.** The weight of line connecting two genes represents the absolute difference in correlation between the ALL group and the AML group. Lines are included in the graphic if the absolute difference in correlation coefficients between the two groups was greater than 0.75. **D) Distribution of the median difference in correlation between ALL and AML for each gene within a module.** The ALL_3 (red) module was not differentially co-expressed according to any of the four methods (PND6, DI, MAD, GHD). The ALL_19 and AML_29 modules (blue) are differentially co-expressed according to at least 3 of the 4 methods and are represented in Figure 6. The ALL_24 module (green) is the module that was only differentially co-expressed according to the PND6 method.
